# Early *Trypanosoma cruzi* Infection Reprograms Human Epithelial Cells

**DOI:** 10.1155/2014/439501

**Published:** 2014-04-09

**Authors:** María Laura Chiribao, Gabriela Libisch, Adriana Parodi-Talice, Carlos Robello

**Affiliations:** ^1^Unidad de Biología Molecular, Montevideo 11400, Institut Pasteur de Montevideo, Uruguay; ^2^Departamento de Bioquímica, Facultad de Medicina, Universidad de la República, Montevideo 11300, Uruguay; ^3^Sección Genética, Facultad de Ciencias, Universidad de la República, Montevideo 11400, Uruguay

## Abstract

*Trypanosoma cruzi*, the causative agent of Chagas disease, has the peculiarity, when compared with other intracellular parasites, that it is able to invade almost any type of cell. This property makes Chagas a complex parasitic disease in terms of prophylaxis and therapeutics. The identification of key host cellular factors that play a role in the *T. cruzi* invasion is important for the understanding of disease pathogenesis. In Chagas disease, most of the focus is on the response of macrophages and cardiomyocytes, since they are responsible for host defenses and cardiac lesions, respectively. In the present work, we studied the early response to infection of *T. cruzi* in human epithelial cells, which constitute the first barrier for establishment of infection. These studies identified up to 1700 significantly altered genes regulated by the immediate infection. The global analysis indicates that cells are literally reprogrammed by *T. cruzi*, which affects cellular stress responses (neutrophil chemotaxis, DNA damage response), a great number of transcription factors (including the majority of NF**κ**B family members), and host metabolism (cholesterol, fatty acids, and phospholipids). These results raise the possibility that early host cell reprogramming is exploited by the parasite to establish the initial infection and posterior systemic dissemination.

## 1. Introduction

*Trypanosoma cruzi*, the causative agent of Chagas disease, has the peculiarity, when compared with other intracellular parasites, to invade almost any type of cell. In* T. cruzi*, pathogen-associated molecular patterns involve a great number of surface molecules that induce changes in cell signaling of host cells [1]. The early cell infection by cell-derived trypomastigotes involves adhesion, penetration, and transit through host cell parasitophorous vacuoles in order to establish an intracellular infection. In this sense, trypomastigotes must interact through their surface with host-surface molecules in order to generate signaling and/or metabolic changes that favor infection. As an obligate intracellular protozoan parasite,* T. cruzi* has evolved several mechanisms for recognition, adhesion, and penetration. Particularly, the presence of hundreds of copies of several gene families coding for surface glycoproteins [2] and the simultaneous expression of several genes of each family [3] are probably responsible for this pathogen's ability to infect a wide range of cell types. However, little is known about the specific responses of each different cell type. Most of the focus has been placed on the study of macrophages and cardiomyocytes, since they are responsible for host defenses and antigen presenting or cardiac lesions in Chagas disease, respectively [4, 5]. However, when these parasites enter their host through a skin lesion, by contact with mucous tissue or by ingestion, the establishment of the infection depends on its ability to rapidly invade epithelial cells that constitute the first barrier against infections. The epithelium provides both a physical barrier and a variety of antimicrobial factors to avoid microbial entry [6]. In this sense, parasites must be able not only to invade epithelial cells, but also to insure dissemination and the establishment of a future chronic infection.

The study of gene expression profiles during infection constitutes a very powerful tool in order to compare global responses of several kinds of cells, allowing the identification of new genes and/or pathways implicated in the establishment of the infection and pathogenesis. Although several reports have been published with these approaches, a high variability in parasite strains, host cells, mammal species, and times of infection generate a complex picture and few general conclusions. Cardiac cells have been the most studied using mice models [7–9], revealing hundreds of differentially expressed genes in infected cells. The response of mice macrophages has also been studied at 24 hours postinfection, comparing different stimuli and cytokine profiles [10]. Recently, Caradonna et al. analyzed the medium and late responses (18 hs and 72 hs, resp.) to* T. cruzi* in HeLa cells, through a different approach (genome-wide RNAi screen [11]) showing the relevance of host metabolism on intracellular* T. cruzi* growth. In this work, we focused our study on the early response of human cells to* Trypanosoma cruzi* infection. It is important to note that previous reports show that the early response of human cells to* T. cruzi* involves minimal modulation of gene expression, particularly in HeLa cells, where few changes were described in the early infection [12, 13]. Epithelial cells were used as a model since, as described above, they constitute the first barrier against infection. As expected, strong changes in gene expression profiling were found immediately after parasites contacted host cells, involving reprogramming of gene expression in the first 6 hours of infection.

## 2. Materials and Methods

### 2.1. Cell Cultures, Parasites, and Infection Assays

HeLa human cell line was grown in Dulbecco's Modified Eagle's Medium (DMEM) (Gibco) supplemented with 10% heat inactivated fetal bovine serum (FBS) (Gibco) at 37°C in a 5% CO_2_ atmosphere. Dm28C* T. cruzi *strain was used throughout this work [14].

For infection assays, cell-derived trypomastigotes were incubated with semiconfluent HeLa cells (10 : 1 parasite : cell ratio) during 4 hours at 37°C in DMEM supplemented with 2% FBS. After interaction period, parasites were removed, and cells were washed with PBS twice and incubated with DMEM with 2% SBF. Cell samples were taken at 0, 3, and 6 hours after the interaction period henceforth named *t*_0_, *t*_3_, and *t*_6_, respectively.

### 2.2. RNA Extraction and Microarray Procedures

Total RNA was isolated with phenol/chloroform as described by the manufacturer (Tri Reagent, Sigma-Aldrich, USA). Processed samples were quantified in a spectrophotometer (NanoDrop 1000 and Thermo Scientific), exhibiting a high content of total RNA and a good quality without degradation, according to the RIN (RNA integrity number) values obtained from a Bioanalyzer 2100 (Agilent Technologies), which were all above 8. Microarray analysis was performed using a 4 × 44 K Human Genome Oligo Microarray (G4112F, Agilent), in a One-color design. A 200 ng aliquot of total RNA was reverse-transcribed into cDNA, and this was transcribed into cRNA and labeled using the Low Input Quick Amp Labeling Kit, One-color (Agilent Technologies). The labeled cRNA was purified with Illustra RNAspin mini Isolation kit (GE Healthcare, USA). The quality of each cRNA sample was verified by total yield and specificity calculated based on NanoDrop ND-1000 spectrophotometer measurements (NanoDrop Technologies, USA).

After that, we proceeded with the hybridization, washing, assembling of the chips, and scanning, according to the protocol specified by Agilent. The glass slides were scanned using an Agilent microarray scanner G2565BA and default settings for all parameters. The labeled samples were placed in human hybridizing chips for 17 h at 60°C with a 10 rpm rotation. Successive washings were done with different washing, stabilization, and drying solutions according to Agilent's Low Input Quick Amp Labeling Kit protocol. We used Agilent Feature Extraction (version 9.5.1) for quality control, data filtering, and data normalization. The software also converts the scanned images in quantitative data for further analysis. The software automatically finds and places microarray grids, rejects outlier pixels, accurately determines feature intensities and calculates log ratios (Agilent's processed signal value), flags outlier pixels, and calculates statistical confidences. It also performs dye normalization within arrays using Lowess normalization. Three biological replicates were performed to each condition.

Microarray experiments were statistically compared using GeneSpring software 12.0 GX. Genes significantly up- and downregulated were identified by the ANOVA-test with a *P* value of 0.05 and a Benjamini-Hochberg false discovery rate correction for multiple testing.

### 2.3. Real-Time RT-PCR

The RNA samples used in the microarray experiment were used to validate some of the differentially expressed genes, through real-time PCR. For each sample, cDNA was synthesized by reverse transcription using the SuperScript II Reverse Transcriptase (Invitrogen) with Oligo(dT) primers and 500 ng of total RNA added as a template. The primer sequences and expected product length of amplicons are listed in Supplementary Table 1 (see Table S1 in Supplementary Material available online at http://dx.doi.org/10.1155/2014/439501). Almost all the primers used span an exon-exon junction to avoid DNA amplification (Supplementary Table 1). Real-time reactions were performed using 5 *μ*L Sybr Green (KAPA SYBR FAST Universal 2X qPCR Master Mix, Kapa Biosystems), 200 nM of forward and reverse primers, and 1 *μ*L of a 1/5 dilution cDNA, in a final volume of 10 *μ*L. Samples were analyzed in duplicate in an Eco real-time PCR System (Illumina). Standard amplification conditions were 3 min at 95°C and 40 cycles of 15 s at 95°C, 30 s at 58°C, and 30 s at 72°C. After each PCR reaction, the corresponding dissociation curves were analyzed to ensure that the desired amplicon was being detected and to discard contaminating DNA or primer dimers.

The threshold cycle (CT) value for each gene was normalized to GAPDH, calculating the ΔCt for each gene in all samples (3 replicates of control and infected cells at *t*_0_). The comparative CT method (ΔΔCt method) was used to determine the relative quantity of the target genes, and the fold change in expression was calculated as 2-ΔΔCt.2,5.

### 2.4. Western Blot

Lysates of control and infected HeLa cells were obtained by washing and resuspending monolayers directly in Cell Lysis Buffer (Promega). After centrifugation at 12000 g for 10 minutes, protein extracts were resolved by SDS-PAGE in a 12% polyacrylamide gel under reducing conditions and electrotransferred to Amersham Hybond ECL Nitrocellulose membranes (GE Healthcare). Membranes were blocked in 5% skimmed milk and 0.1% Tween 20 in PBS for 1 hour at 22°C. After washing with 0.1% Tween PBS, blots were incubated with an appropriate dilution of primary antibody, overnight at 4°C, in 1% Bovine Serum Albumin (BSA) (Sigma) and 0.1% Tween 20 in PBS. After three washes, a dilution of peroxidase conjugated anti-mouse antibody (DAKO) was applied at room temperature for 1 hour. The signal was developed with Super Signal West Pico Chemiluminiscent Substrate (Thermo Scientific).

### 2.5. Red Nile Staining

For Red Nile staining assay, cells were seeded in 12-well plates with coverslips and infected with trypomastigotes for 4 hours. After 0, 3, and 6 hours (*t*_0_, *t*_3_, and *t*_6_), cells were fixed with 4% paraformaldehyde for 20 minutes at room temperature. After fixation, coverslips were incubated with 0.1 M glycine for 10 minutes and permeabilized with 0.5% Triton-X100 for 5 minutes at room temperature. For neutral lipid staining, cells were incubated for 10 minutes at 37°C with 500 nM Red Nile (Sigma) and rinsed with PBS. Coverslips were mounted with Fluoroshield with DAPI (Sigma) and visualized in a LeicaTCSSP5 Confocal Microscope.

## 3. Results

### 3.1. *Trypanosoma cruzi* Early Infection Remodels HeLa Cell Gene Expression

**Figure 1:**
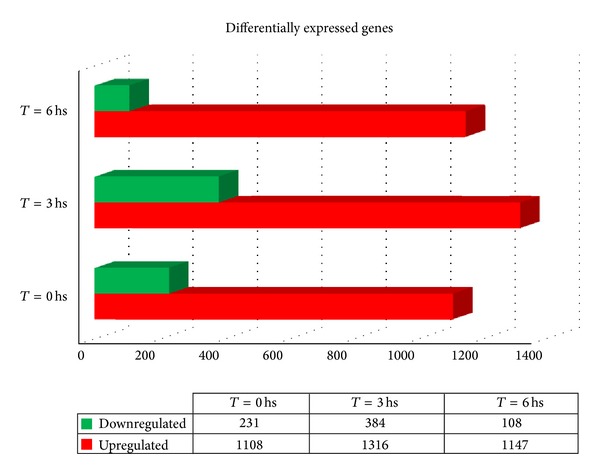
Differential gene expression in* T. cruzi *infected HeLa cells at *t*_0_, *t*_3_, and *t*_6_ compared to noninfected control cells (≥2-fold, *P* ≤ 0.05). Red bars indicate upregulated genes and green bars indicate downregulated genes. Inset table shows number of differentially expressed genes for each condition.

**Figure 2:**
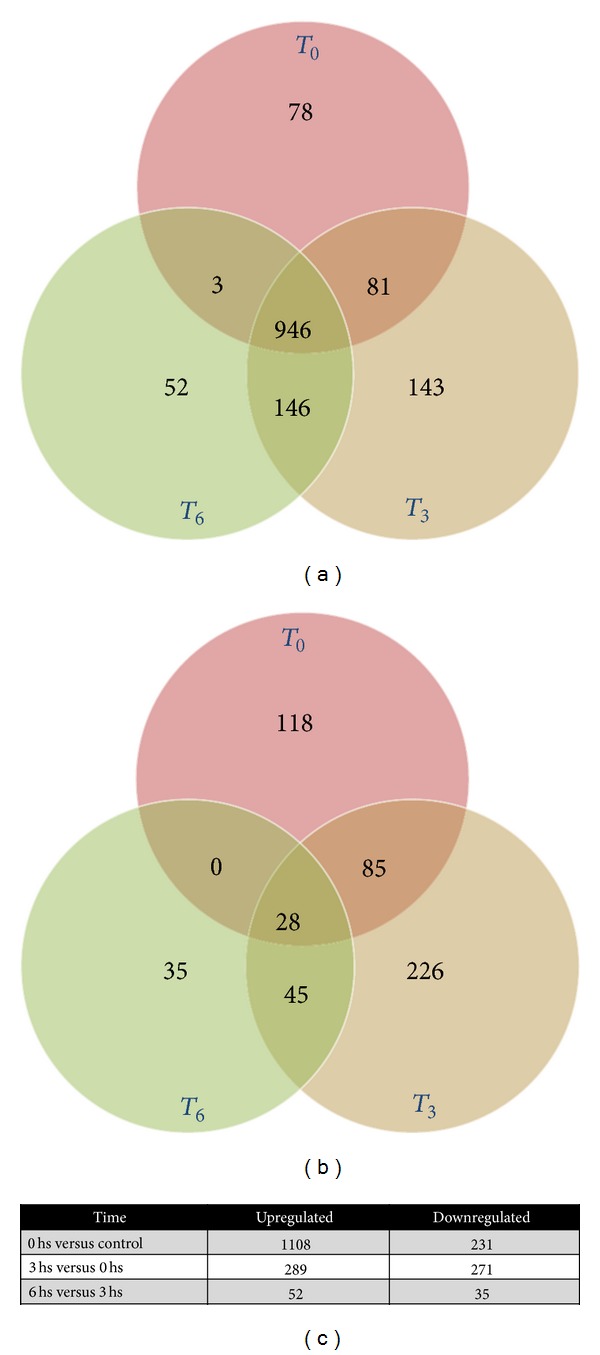
Venn diagrams comparing (a) upregulated and (b) downregulated genes (fold change ≥ 2, *P* ≤ 0.05) with respect to control cells; (c) number of genes up- or downregulated, comparing one condition to the previous one (*t*_0_ versus C, *t*_3_ versus *t*_0_, and *t*_6_ versus *t*_3_) using fold change ≥ 2 and *P* ≤ 0.05.

The effect of* T. cruzi *early infection on host gene expression was investigated on infected HeLa cells at 0, 3, and 6 h postinteraction period (*t*_0_, *t*_3_, and *t*_6_), representing the initial events of penetration, intravacuolar stage, and the release of parasites to the cytosol, respectively, as previously described [9]. Total RNA extracted from infected and noninfected cells was labeled and hybridized to a Human GE 4 × 44 K Microarray (Agilent), which allows the evaluation of the gene expression profile of 19,596 different human genes. Genes showing at least a 2-fold change in their expression and a 95% probability of being differentially expressed (*P* < 0.05) were considered to be significantly regulated by the infection. The total number of significant differentially expressed genes is shown in Figure 1: more than a thousand genes are upregulated in the early response to infection, whereas less than 400 genes were downregulated, when comparing control versus* T. cruzi* infected cells. Major changes were observed at 3 hr postinfection, with a total of 1700 differentially expressed genes. During the course of the early response to the infection most of the upregulated genes (946) changed at *t*_0_ and remained in this condition in all further times (Figure 2(a)). In contrast, only 28 genes remained downregulated during the 6-hour period (Figure 2(b)). A selection of 300 upregulated and 100 downregulated genes is presented in Supplementary Table 2.

### 3.2. Gene Ontology (GO) and Pathway Analysis

**Figure 3:**
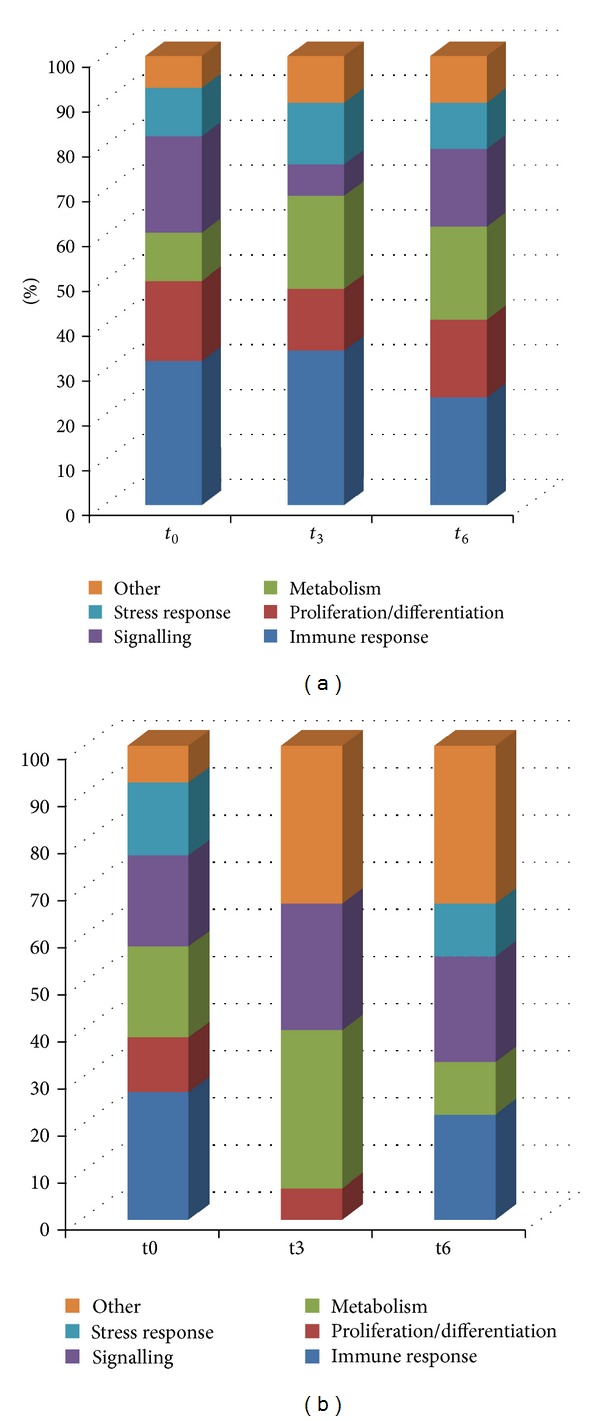
Cellular processes predicted to be modulated during* T. cruzi* infection. (a) Pathways analysis with upregulated genes from *t*_0_, *t*_3_, and *t*_6_; (b) pathways analysis with upregulated genes comparing *t*_0_ versus C, *t*_3_ versus *t*_0_, and *t*_6_ versus *t*_3_, showing characteristic pathways altered at each time point.

**Figure 4:**
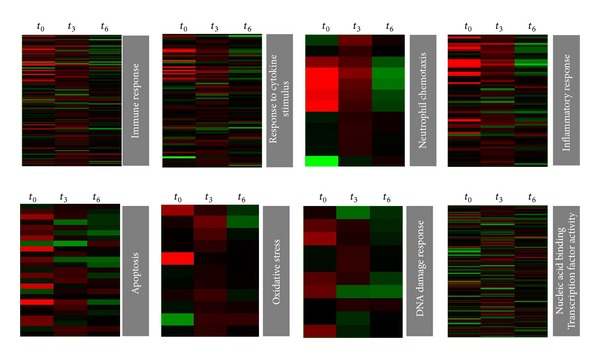
Heat maps of some representative biological processes altered during* T. cruzi* infection. Genes regulated by* T. cruzi* involved in immune response/inflammation (top) and genes involved in stress response and transcription factor activity (down) are represented.

**Figure 5:**
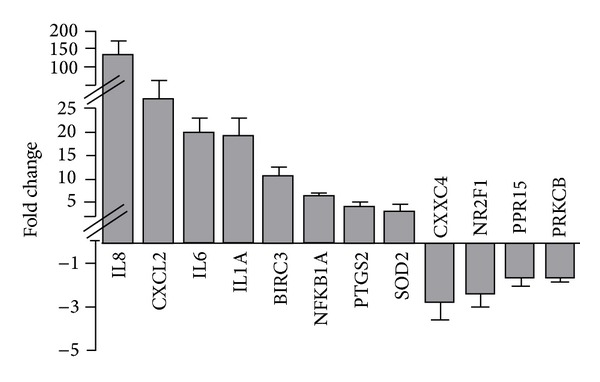
Quantitation of selected genes by real-time PCR. Mean relative fold changes of upregulated and down-egulated genes analyzed by qPCR in 3 biological replicates of infected HeLa cells at *t*_0_ relative to that in control cells. Error bar represents SD of replicates. The cross-bars in the *y*-axis indicate changes in the scale used.

GO and pathway analysis were performed using GeneSpring (Agilent Technologies), comparing each time point with control cells, indicating that a wide range of biological processes was altered immediately after infection, the most relevant ones being immune response, cellular defense mechanisms, proliferation/differentiation, metabolism, and cell signaling (Figure 3(a)). However, at each stage of the early infection the most affected pathways differ significantly when comparing each condition with the previous one (Figure 3(b)). Hence the major expression changes in the proinflammatory response take place at *t*_0_ (e.g., Toll-like receptor pathway and TNF-*α* and TGF-*β* signaling), and the remodeling of metabolism is maximal at *t*_3_ (e.g., folate metabolism and lipid metabolism), whereas at *t*_6_ the highest changes tend towards transport processes and stress response other than immune responses (e.g., DNA damage response and signaling by G protein receptors). An overview of the most affected pathways is shown in Figure 4. Representative genes of the affected pathways were further evaluated by real-time PCR (Figure 5), confirming these results.

As previously reported [7, 9, 12, 15], infection of mammalian cells (other than epithelial ones) by* T. cruzi *elicits a strong response whose major contribution is due to interrelated inflammatory, apoptotic, stress, and proliferative responses. In the present model of early response of epithelial cells we found that more than 50% of the affected pathways were related to these processes, mainly at *t*_0_ (Figures 3 and 4). The intensity of this response is more evident when only those genes that are upregulated more than 10 times with respect to the control are grouped (Table 1).

### 3.3. The Predominance of Neutrophil Chemotactic Factors

With respect to inflammation related genes, most of them are presented in Table 1: namely, they are highly overexpressed and, as expected, major changes occur in cytokines and chemokines. Remarkably, the most upregulated chemokines have similar functions: recruitment of professional phagocytic cells (CXCL1, CXCL2, and IL-8), particularly neutrophils. Besides, the common receptor for some of them (CXCR2) is also overexpressed. It is worth mentioning that IL-8 is a chemotactic factor rather than a classical cytokine (currently named CXCL8) and was discovered as “neutrophil chemotactic factor” [16].

### 3.4. Regulation of Genes Controlling Cell Survival

Concerning programmed cell death, the predominance of a high number of genes related to inhibition of apoptosis is notorious. In particular, the antiapoptotic genes BIRC3 at *t*_0_ and BCL2A1 at *t*_3_ reach a maximum of 23-fold and 27-fold increase on their expression, respectively, and they remain highly overexpressed along the early response. The proliferative response is more relevant at *t*_0_ and the main pathways involved are TNF-like weak inducer of apoptosis (TWEAK) signaling pathway, which relates the inflammatory and proliferative responses through NF*κ*B signaling. The Wnt signaling pathway is also significantly altered during early infection (Supplementary Table 3). It is noteworthy that, among the proliferative response related genes, there is a synergism with the highly induced chemokines: the colony stimulating factor 3, specific for neutrophils, is sevenfold upregulated immediately after infection (Supplementary Table 2).

### 3.5. Transcription Factors in the Early Response to Infection

Many of the differentially regulated genes following infection were transcription factors whose function affects the expression of many genes (see specific heat map in Figure 3). In Supplementary Table 4 a list of the up- and downregulated transcription factors is presented: as can be seen, several members of the NF*κ*B family (Table 2) change their expression, suggesting activation by different pathways. Particularly RELB, related to the noncanonical pathway, is among the most upregulated genes. Several members of adaptor-related protein complex 1 (AP-1) family proteins were also upregulated, such as JUN, JUND, ATF2, FOSL1, and FOSL2. These transcription factors regulate a variety of activities including proliferation, apoptosis, and inflammation in response to different stress signals including microbial infections [17]. Another remark is that 15% of the downregulated genes are transcription factors.

### 3.6. Induction of DNA Damage Response Related Genes

**Figure 6:**
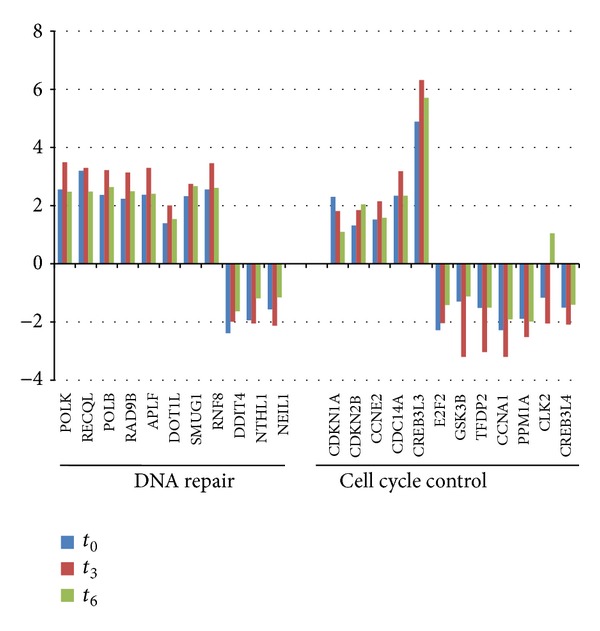
Temporal regulation of host genes related to cell cycle control and DNA repair on infected cells.

The expression of genes involved in DNA damage response (DDR) pathways was significantly regulated by infection. DDR is a highly coordinated cellular system able to sense and counteract DNA damage caused by a variety of environmental and endogenous genotoxic insults [18]. Among all the genes related to DDR transduction pathways, we grouped those involved in DNA repair mechanisms and those related to cell cycle regulation (Figure 6). Among DNA repair-related genes, there are several encoding for enzymes involved in DNA metabolism that were induced upon infection such as polymerase (DNA directed) kappa (POLK), polymerase (DNA directed) beta (POLB), RecQ protein-like (DNA helicase Q1-like) (RECQL), and single-strand-selective monofunctional uracil-DNA glycosylase 1 (SMUG1). We also detected differentially expressed genes that participate in recruitment of mediators or effectors of DNA repair such as promyelocytic leukemia (PML), DOT1-like histone H3K79 methyltransferase (DOT1L) and ring finger protein 8, and E3 ubiquitin protein ligase (RNF8). Several genes involved in cell cycle control were found to be modulated after infection. Among these, p15 (CDKN2B) and p21 (CDKN1A), which are cyclin-dependent kinase inhibitors implicated in the suppression of cell proliferation under several stresses, were upregulated upon infection.

### 3.7. *Trypanosoma cruzi* Alters Host Cell Lipid Metabolism

**Figure 7:**
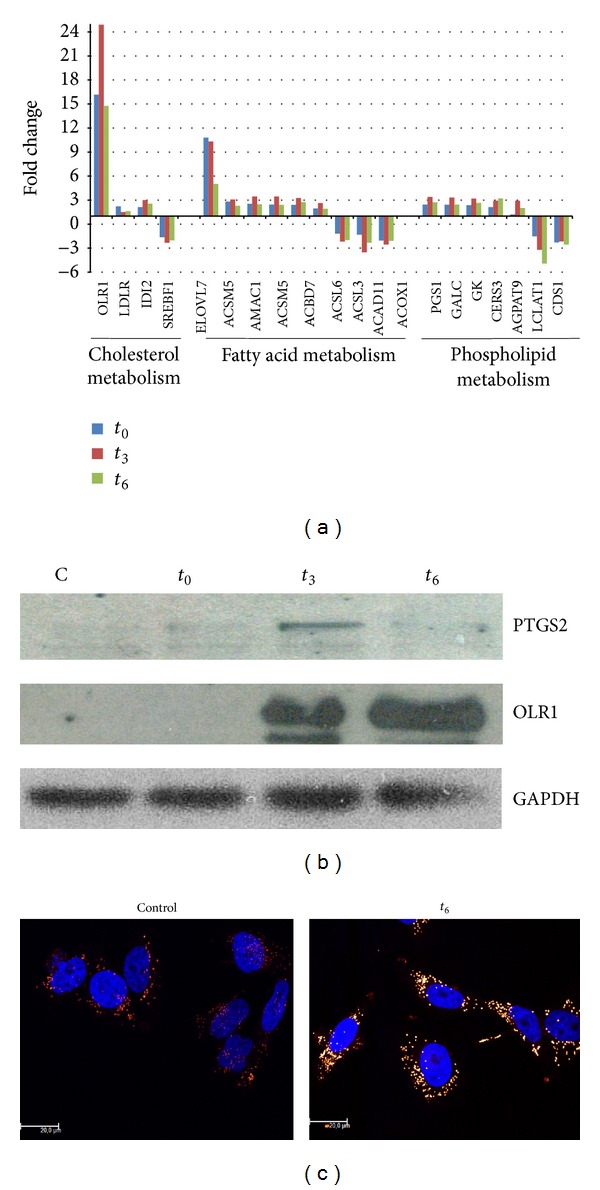
(a) Temporal regulation of host genes related to lipid metabolism after infection with* T. cruzi*. (b) Western blot analysis showing overexpression of PTGS2 and OLR1 proteins and normalization with GADPH. (c) Red Nile staining of control and infected cells at *t*_6_.

A group of host genes significantly regulated by* T. cruzi* were those involved in host lipid metabolism: cholesterol, fatty acids, and phospholipids (Figure 7(a)). Concerning cholesterol metabolism, the most remarkable changes occur in cholesterol transport related genes: low density lipoprotein receptor (rLDL) and oxidized low density lipoprotein receptor (OLR1), responsible for the entry of cholesterol and oxidized cholesterol, respectively, are overexpressed immediately after infection. In particular, OLR1 gene expression is highly upregulated, not only at the transcriptional level (Table 1) but also at the translational level (Figure 7(b)). ABCA1, which mediates the cholesterol flux and has been described to be overexpressed in cells that accumulate cholesterol [19], is also upregulated upon infection. Several genes from fatty acid metabolism are affected, in particular members of the acyl-CoA synthetase family (ACSL6, ACSM5, and AMAC1; Figure 7(a)) and fatty acids transport (SLC27A1) that participate in fatty acid activation and uptake [20]. One of the most overexpressed genes (Table 1) is a very long chain fatty acid, elongase 7 (ELOVL7), which elongates fatty acids of 16 to 24 carbons, with the highest activities towards C18-CoAs [21, 22]. Very long fatty acids are essential precursors of signaling molecules related to the arachidonic acid and prostaglandin metabolisms, the limiting pathway being catalyzed by prostaglandin-endoperoxide synthase 2 (PTGS2). Consistently, we found that this gene is rapidly overexpressed both at the transcriptional and translational levels (Figure 7(b) and Supplementary Table 2). These changes were so drastic in host cells to the extent that, six hours after penetration, a significant accumulation of lipid bodies was found [23] (Figure 7(c)).

## 4. Discussion

The ability of* Trypanosoma cruzi* to invade almost any kind of nonimmune cell makes Chagas a complex parasitic disease in terms of prophylaxis and therapeutics. Since the parasite cannot go through the skin, penetration is possible through the site of the insect bite (mainly after scratching the skin) or by invading mucous tissues (this would include cases of oral infection). In any case, the common physical barrier is constituted by epithelial cells, and the early infection will be relevant for the establishment of a chronic disease. Some reports about transcriptomics of the human cell response in epithelium and fibroblasts showed minimal alterations at the level of gene expression. De Avalos et al. [13] described that only 6 genes were downregulated, whereas no genes were induced in the fibroblast early response to* T. cruzi* infection. On the other hand, in epithelial cells only 41 genes were found to be induced after infection [12]. However, in a more recent work in mice cardiomyocytes [9] the authors made a very complete characterization of the cell response and showed that hundreds of genes changed, a result that is expected for such a complex intracellular parasite. In that context we decided to evaluate the early response to* T. cruzi* by analyzing three time points (*t*_0_, *t*_3_, and *t*_6_), in order to get a general view of the response during adhesion/penetration, the intravacuolar stage, and the early cytosolic stage, respectively. The results of this study demonstrated that immediately after contact with human epithelial cells,* T. cruzi* triggers a strong response, where more than 1300 genes were upregulated and almost 400 genes were downregulated. Most of the regulated genes are involved either in cellular defense, metabolism, or response to stress, including DNA damage response. Some genes of our interest were further evaluated by real-time PCR, and two inflammation related genes were studied at protein level, showing that mRNA upregulation was correlated with protein induction. The major response was achieved at *t*_0_, and most genes (946) were maintained upregulated during early infection, whereas only 28 of the downregulated genes were common to the three studied time points.

### 4.1. Cellular Defense Mechanisms

In this work, a number of genes involved in cellular defense mechanisms were found differentially expressed, particularly genes related to inflammation and immune response. This response is characteristic in Chagas disease, and the fact that about 30% of the altered genes belong to this category is not surprising. However, the pattern of chemical mediators differed substantially from previous gene profiling studies involving* T. cruzi*. The majority of the most highly expressed chemokines function as chemotactic factors for neutrophils. Particularly, the most upregulated gene is IL-8 (327- and 157-fold at *t*_0_ in microarray experiment and RT-PCR, resp.). As was previously described, this molecule is a potent neutrophil chemotactic factor, belonging to the CXC chemokine subfamily, which signals through CXCR1 and CXCR2 G-coupled proteins. The two major effects of IL-8 are chemotaxis of neutrophils and neovascularization. Moreover, it enhances the proliferation and survival of endothelial cells and angiogenesis [24]. This property has been related to the ability of cancer cells to survive and migrate from the primary site [25]. Although this proinflammatory response clearly reflects defense mechanisms against the parasite, we cannot discard that it can be exploited by the parasite as a strategy for survival and dissemination: vascularization and recruitment of cells that can be infected by* T. cruzi*. This strategy for dissemination by PMN attraction and infection has been reported for* Leishmania* infection, mainly through overexpression of IL-8 [26], and also for many intracellular pathogens that can survive in the hostile neutrophil-filled environment, and this enables the subsequent infection of macrophages [27]. CXCR2, the major receptor for IL-8, is also clearly overexpressed, indicating that this chemokine also acts on HeLa cells. Interestingly, IL-8 binding to CXCR2 triggers the rise of intracellular calcium from intracellular stores [28], which may in turn contribute to infection.

An important feature in the infection by* T. cruzi* is the oxidant stress response that is very relevant for the parasite's pathogenesis. Although the oxidative stress generated after the infection is a hallmark of professional phagocytic cells, it has been demonstrated that oxidative stress in nonphagocytic cells is generated by many mechanisms, including dysfunction of mitochondria in infected mice [29] and cardiomyocytes [30] and cytokines signaling like TNF [5], and also by the action of lipoxygenase, cyclooxygenase, and cytochrome P450-dependent oxygenases during arachidonic acid metabolism [31]. In infected epithelial cells we found many enzymes involved in arachidonic acid metabolism and many p450-dependent oxygenases as well as TNF alpha-signaling pathway activity, which suggests that many sources of ROS are active in these cells. Consistent with this idea, many antioxidant enzymes that are known to be upregulated after oxidative stress, particularly mitochondrial superoxide dismutase (SOD2), glutamate-cysteine ligase (GCLC) and hemoxygenase 1 (HMOX1), metallothioneins (MTX), gluthathione peroxidase (GPX2), and thioredoxin reductase 1 (TXNRD1), are induced after infection in epithelial cells [32]. It has been proved that exposure to TNF leads to an acute GSH depletion in epithelial cells due to its oxidation to GSSG. The reduction of GSH is sensed by stressed cells mainly through redox-sensitive transcription factors (AP-1 and Nf*κ*B), causing phosphorylation and degradation of I*κ*B, which is a critical step for Nf*κ*B activation [33, 34]. On the other hand, the activation of NF*κ*B regulates the expression of many proinflammatory cytokines like IL-8, IL-6, and TNF *α*, as well as antioxidant enzymes [35]. Due to the interconnection among inflammation and oxidative stress, it seems that the appropriate balance between proinflammatory and prooxidant and antioxidant and anti-inflammatory mediators could be responsible for the overall response in Chagas disease infection [36, 37]. As in infected cardiomyocytes [38], this balance could also determine the activation of DDR seen in infected epithelial cells and also the resolution of these pathways leading either to senescence, DNA repair, or apoptosis.

### 4.2. *Trypanosoma cruzi* Early Infection Regulates Genes Involved in Metabolism

Genes involved in cellular metabolism were found to be regulated during early* T. cruzi* infection. This is a common observation in host-pathogen interactions, although in the case of* T. cruzi*, metabolism remodeling has been widely associated with the generation of ATP by host cells [11]. In this study, a strong component of lipid metabolism was found, mainly affecting genes related to cholesterol, fatty acid, and phospholipid metabolism. Remarkably, OLR1 is one of the most upregulated genes and its expression at the translational level indicates that the expression of the receptor peaks at 3 hours after invasion. In* T. cruzi*, it was previously reported that infection enhances LDL receptor expression, which is used by the parasite to enter host cells [39]. We also found upregulation of this receptor gene but at a lower level, whereas OLR1 is more than 15-fold overexpressed along the experiment when compared to noninfected cells. It has been demonstrated that* Chlamydia pneumoniae* induces overexpression of OLR1, which in turn serves as a gateway for* Chlamydia pneumoniae* invasion [40–42]. The finding of a strong upregulation of OLR1 deserves further studies in order to evaluate whether it can constitute a new strategy of* T. cruzi* invasion. Finally, since OLR1 is a relevant factor in the development of atherosclerotic lesions, this strong upregulation during* T. cruzi* infection can explain, at least in part, the major susceptibility to atherosclerosis in* T. cruzi* infected mice [43].

Concerning fatty acid metabolism, it was recently described that, during the medium and late* T. cruzi* infection of HeLa cells, perturbations in host fatty acid metabolism alter intracellular parasite growth rates [11]. In particular, Caradonna et al. showed that the genes coding for proteins involved in activation/transport of VLCFAs (SLC27A2) and *β*-oxidation (ACAA1 and IDH1) in peroxisomes directly affect intracellular amastigotes growth. Interestingly, here we found that SLC27A1 gene is overexpressed in infected cells. SLC27A1 codes for a long chain fatty acid (LCFA) transporter,  located in the plasma membrane. Taken together, these results allow us to hypothesize that both events are connected: during the early infection parasites favor LCFA cell uptake and elongation of LCFA to VLCFA through ELOV7 overexpression. These VLCFAs will be used in peroxisomes for *β*-oxidation during the medium and late infection [44]. However, it should be remarked that energy production is not the most important role of VLCFAs: they are relevant for many physiological processes like membrane composition maintenance, inflammatory responses, and neutrophil migration, as well as in the synthesis of signaling molecules like eicosanoids, sphingosine 1-phosphate, and precursors of prostaglandins biosynthesis [21, 22, 45]. In this sense, the induction of PTGE2 in the early infection suggests a coordinated function with ELOVL7. Which is the relative relevance of the different roles of VLCFAs with respect to the parasite survival and establishment of the infection remains to be elucidated.

Finally, several genes coding for phospholipid metabolism related enzymes were regulated under infection, such as CER3, which is essential for epidermal lipid homeostasis [46], AGPAT9, and PGS1, while LCLAT1 and CDS1 were downregulated, suggesting the relevance of phospholipid metabolism in signaling and membrane homeostasis during* T. cruzi* early infection. All of these results led us to evaluate the presence of lipid bodies in the immediate response of HeLa cells to* T. cruzi*. Lipid bodies constitute lipid rich organelles involved in cell metabolism and signaling and also a nutrient source for intracellular pathogens [47]. They are highly regulated in macrophages derived from mice infected with* T. cruzi* leading to foamy cell formation and, in some cases, to atherosclerotic lesions [48]. In this work we demonstrate the formation of lipid accumulation immediately after infection, indicating that this is a more general parasite strategy, not only confined to macrophages.

### 4.3. *Trypanosoma cruzi* Early Infection Induces DNA Damage Response

DDR is a signaling network triggered upon DNA lesions, which coordinates several programs, including DNA repair, cell cycle checkpoints, senescence, or apoptosis [18]. The DDR components in the transduction pathway are sensors of damage, signal transducers, and effectors. DNA damage leads to activation of ataxia-telangiectasia mutated (ATM) and ATM- and Rad3-related (ATR) signaling pathways, depending on the nature of the damage. These kinases, in turn, phosphorylate multiple substrates to activate cell cycle regulators and stimulate DNA repair by inducing DNA-repair proteins transcriptionally or posttranscriptionally. In this study, host genes involved in DDR were found to be significantly regulated upon infection with* T. cruzi*. Among them, several genes encoding for enzymes involved in DNA metabolism were induced upon infection, such as DNA polymerases POLK and POLB, DNA helicase RECQL, and DNA glycosylase SMUG1 involved in various types of DNA repair, including mismatch repair, base excision repair, and direct repair. In addition to DNA repair-related genes, we also detected differentially expressed genes that participate in the recruitment of mediators or effectors of DNA repair, such as PML, DOT1L, and RNF8. Interestingly, PML (promyelocytic leukemia protein), involved in chromatin metabolism and DNA repair, has been associated with response to viral infection [49].

On the other hand, a group of DDR related genes which contribute to cell cycle arrest were altered in the early infection (Figure 6). It is remarkable that p21 (CDKN1A) is upregulated in the early infection, suggesting that* T. cruzi* infection triggers a transient cell cycle arrest which may allow the cell to repair the damage. In addition to CDKN1A, other genes detected as differentially expressed have been involved in cell cycle progression regulation at G1, such as CDKN2B, CCNE2, CLK2, GSK3B, PPP2R5C, E2F2, PPM1A, TFDP2, CCNA1, and DCD14A. The rapid induction of DDR leads to the need to develop further studies to confirm whether DNA damage occurs immediately after infection. Besides, taking into account that DDR involves not only gene expression changes, but also posttranslational modifications, mainly due to the kinase activities of ATM and ATR, a profound evaluation of this response should be performed.

## 5. Conclusions

In summary, we have demonstrated that the early response to* T. cruzi* infection has a widespread effect on the expression of host genes involved in cellular defense mechanisms, stress responses, and metabolism. The importance of these findings is also relevant by comparing our findings to those previously reported in HeLa cells. The gene expression patterns identified may also provide insights into some of the strategies of initial infection, particularly dissemination: we postulate similar mechanisms of metastatic cancer cells mediated by interleukin 8. Finally, we show activation of DNA damage response, which deserves further studies.

## Supplementary Material

Supplementary Table 1: Primers used for real time PCR.
Supplementary Table 2: Gene expression changes in *T. cruzi* infected HeLa at t_0_, t_3_ and t_6_ compared to control cells (≥ 2-fold, *p* ≤ 0.05).
Supplementary Table 3: Pathways analysis of upregulated genes (fold change ≥ 2, *p* ≤ 0.05) with respect to control cells at A) t_0_, B) t_3_ and C) t_6_.
Supplementary Table 4: List of transcription factors modulated in host cells by *T. cruzi* infection.

## Figures and Tables

**Table 1:** Overexpressed genes with fold change ≥10 at any time postinfection (*P* ≤ 0.05).

Gene ID	Description	FC-*t*_0_	FC-*t*_3_	FC-*t*_6_
Immune response				
CCL20	Chemokine (C-C motif) ligand 20	85,2	93,3	14,5
CCL8	Chemokine (C-C motif) ligand 8	39,9	23,1	3,1
CD96	CD96 molecule	27,4	24,4	24,2
CXCL1	Chemokine (C-X-C motif) ligand 1	20,0	18,9	7,6
CXCL2	Chemokine (C-X-C motif) ligand 2	49,8	12,3	6,2
CXCL3	Chemokine (C-X-C motif) ligand 3	64,9	12,7	4,1
EREG	Epiregulin	9,2	13,8	4,1
IL1A	Interleukin 1, alpha	18,0	6,3	2,3
IL6	Interleukin 6	27,3	6,9	2,6
IL8	Interleukin 8	326,9	67,9	16,6
OLR1	Oxidized low density lipoprotein (lectin-like) receptor 1	16,2	24,9	14,7
PTX3	Pentraxin 3, long	13,8	13,1	9,3
Apoptosis inhibition				
BCL2A1	BCL2-related protein A1	16,4	27,4	8,2
BIRC3	Baculoviral IAP repeat containing 3	23,6	7,2	2,8
Transcription				
LMO2	LIM domain only 2 (rhombotin-like 1)	11,6	12,4	4,6
MYCL1	V-myc myelocytomatosis viral oncogene homolog 1	2,2	5,5	10,7
RELB	V-rel reticuloendotheliosis viral oncogene homolog B	11,0	9,3	5,0
Signalling				
RGR	Retinal G protein coupled receptor	20,7	24,1	20,4
PRKG1	Human mRNA for type I beta cGMP-dependent protein kinase	22,2	27,7	24,0
Binding				
MUC4	Mucin 4, cell surface associated	1,9	10,7	4,5
SDC4	Syndecan 4	11,4	7,7	2,5
STATH	Statherin	10,7	13,2	10,9
Transport				
SLC9A6	Solute carrier family 9 (sodium/hydrogen exchanger), member 6	12,7	13,7	11,3
Fatty acid synthesis				
ELOVL7	ELOVL fatty acid elongase 7	10,8	10,3	5,1
Stress response				
SOD2	Superoxide dismutase 2, mitochondrial	15,2	3,3	3,2
Other				
TCL6	T-cell leukemia/lymphoma 6 (nonprotein coding)	28,4	28,3	26,1
TNIP3	TNFAIP3 interacting protein 3	19,4	28,9	10,6
WFDC10A	WAP four-disulfide core domain 10A	3,2	10,0	10,1
NCRNA00246A	nonprotein coding RNA 246A	10,0	11,9	11,3
LOC283174	Hypothetical LOC283174	44,3	39,9	40,4
FLJ44715	cDNA FLJ44715 fis	18,8	21,0	19,6
A1BG	cDNA FLJ31639 fis	29,1	39,1	30,8
ART3	ADP-ribosyltransferase 3	2,2	5,8	12,5

**Table 2:** Members of NF*κ*B family differentially expressed during infection with *T. cruzi*.

Gene	FC-*t*_0_	FC-*t*_3_	FC-*t*_6_
NKFB1	4,0	2,2	1,4
NFKB2	4,1	3,7	2,2
REL	2,5	1,3	−1,0
RELB	11,0	9,3	5
NFKBIA	4,2	1,3	1,5
NFKBIE	3,2	1,7	1,8
NFKBIZ	4,2	1,2	1,8
