# Supplementary material for: Early Trypanosoma cruzi Infection Reprograms Human Epithelial Cells
Source: Biomed Res Int. 2014 Apr 9;2014:439501. doi: 10.1155/2014/439501 (PMC4000934; doi:10.1155/2014/439501)
Supplement: Supplementary file 1 — Supplementary Table 1: Primers used for real time PCR. Supplementary Table 2: Gene expression changes in T. cruzi infected HeLa at t0, t3 and t6 compared to control cells (≥ 2-fold, p ≤ 0.05). Supplementary Table 3: Pathways analysis of upregulated genes (fold change ≥ 2, p ≤ 0.05) with respect to control cells at A) t0, B) t3 and C) t6. Supplementary Table 4: List of transcription factors modulated in host cells by T. cruzi infection. [file 439501.f1.docx]

**Supplementary Table 1** Primers used for real time PCR

| **Gen** | **Primer 5´-3´** | **Product lenght (pb)** | **Span Exon –exon junction?** |
| --- | --- | --- | --- |
|  |  |  |  |
| **IL-8-F** | CACCGGAAGGAACCATCTCA | **110** | **yes** |
| **IL-8-R** | TGGCAAAACTGCACCTTCACA |  |  |
|  |  |  |  |
| **Il1-A F** | TCACGGCTGCTGCATTACAT | **179** | **yes** |
| **IL1A-R** | GGGTATCTCAGGCATCTCCTTC |  |  |
|  |  |  |  |
| **IL-6-F** | TTCGGTCCAGTTGCCTTCTC | **193** | **yes** |
| **IL-6-R** | TACATGTCTCCTTTCTCAGGGC |  |  |
|  |  |  |  |
| **BIRC-3-F** | GGCAGCAGGTTTACAAAGGAG | **143** | **yes** |
| **BIRC3-R** | ACCCATGCACAAAACTACCTC |  |  |
|  |  |  |  |
| **NFKBIA-F** | TGCACTTGGCCATCATCCAT | **190** | **no *** |
| **NFKBIA-R** | TCTCGGAGCTCAGGATCACA |  |  |
|  |  |  |  |
| **CXCL2-F** | ACGGCAGGGAAATGTATGTGT | **231** | **no** |
| **CXCL2-R** | TCGAAACCTCTCTGCTCTAACA |  |  |
|  |  |  |  |
| **SOD2-F** | CACTGCAAGGAACAACAGGC | **201** | **yes** |
| **SOD2-R** | GGGATCATTAGGGTATGATCAGCA |  |  |
|  |  |  |  |
| **PTGS2-F** | CAAATTGCTGGCAGGGTTGC | **139** | **yes** |
| **PTGS2-R** | AGGGCTTCAGCATAAAGCGT |  |  |
|  |  |  |  |
| **PRKCB-F** | CAAAAGCTTGTGGGCGAAAT | **227** | **yes** |
| **PRKCB-R** | CACCACAATAGCCGTTGAGC |  |  |
|  |  |  |  |
| **CXXC4-F** | GCTCATCAACTGTGGCGTCT | **120** | **yes** |
| **CXXC4-R** | GGTGTTCTCTCTAGTGAAGTGC |  |  |
|  |  |  |  |
| **PRR15-F** | TTAAGCCCGAGCAGCACAG | **222** | **yes** |
| **PRR15-R** | TCTGCTGTTGGTGAGCGATT |  |  |
|  |  |  |  |
| **NR2F1-F** | CCAGAGCCAGCAGCACAT | **242** | **yes** |
| **NR2F1-R** | CTCGCTGAACCGCTTCCC |  |  |

**Supplementary Table 2.** Gene expression changes in *T. cruzi* infected HeLa at t_0_, t_3_ and t_6_ compared to control cells ((≥ 2-fold, p ≤ 0.05).

| **Gene symbol** | **FC- t_0_** | **FC- t_3_** | **FC -t_6_** | **Description** |
| --- | --- | --- | --- | --- |
| **Upregulated genes** | | | | |
| IL8 | 326,9 | 67,9 | 16,6 | interleukin 8 (IL8), mRNA [NM_000584] |
| CCL20 | 85,2 | 93,3 | 14,5 | chemokine (C-C motif) ligand 20 (CCL20), transcript variant 1, mRNA [NM_004591] |
| CXCL3 | 64,9 | 12,7 | 4,1 | chemokine (C-X-C motif) ligand 3 (CXCL3), mRNA [NM_002090] |
| CXCL2 | 49,8 | 12,3 | 6,2 | chemokine (C-X-C motif) ligand 2 (CXCL2), mRNA [NM_002089] |
| LOC283174 | 44,3 | 39,9 | 40,4 | hypothetical LOC283174 (LOC283174), non-coding RNA [NR_024344] |
| CCL8 | 39,9 | 23,1 | 3,1 | chemokine (C-C motif) ligand 8 (CCL8), mRNA [NM_005623] |
| A1BG | 29,1 | 39,1 | 30,8 | cDNA FLJ31639 fis, clone NT2RI2003655, weakly similar to ZINC FINGER PROTEIN 195. [AK056201] |
| TCL6 | 28,4 | 28,3 | 26,1 | T-cell leukemia/lymphoma 6 (non-protein coding) (TCL6), non-coding RNA [NR_028288] |
| CD96 | 27,4 | 24,4 | 24,2 | CD96 molecule (CD96), transcript variant 1, mRNA [NM_198196] |
| IL6 | 27,3 | 6,9 | 2,6 | interleukin 6 (interferon, beta 2) (IL6), mRNA [NM_000600] |
| BIRC3 | 23,6 | 7,2 | 2,8 | baculoviral IAP repeat containing 3 (BIRC3), transcript variant 1, mRNA [NM_001165] |
| PRKG1 | 22,2 | 27,7 | 24,0 | Human mRNA for type I beta cGMP-dependent protein kinase (EC 2.7.1.37). [Y07512] |
| RGR | 20,7 | 24,1 | 20,4 | retinal G protein coupled receptor [Source:HGNC Symbol |
| CXCL1 | 20,0 | 18,9 | 7,6 | chemokine (C-X-C motif) ligand 1 (melanoma growth stimulating activity, alpha) (CXCL1), mRNA [NM_001511] |
| TNIP3 | 19,4 | 28,9 | 10,6 | TNFAIP3 interacting protein 3 (TNIP3), transcript variant 1, mRNA [NM_024873] |
| FLJ44715 | 18,8 | 21,0 | 19,6 | cDNA FLJ44715 fis, clone BRACE3021430. [AK126671] |
| IL1A | 18,0 | 6,4 | 2,6 | interleukin 1, alpha (IL1A), mRNA [NM_000575] |
| BCL2A1 | 16,4 | 27,4 | 8,2 | BCL2-related protein A1 (BCL2A1), transcript variant 1, mRNA [NM_004049] |
| OLR1 | 16,2 | 24,9 | 14,7 | oxidized low density lipoprotein (lectin-like) receptor 1 (OLR1), transcript variant 1, mRNA [NM_002543] |
| SOD2 | 15,2 | 3,3 | 3,2 | superoxide dismutase 2, mitochondrial, mRNA (cDNA clone MGC:21350 IMAGE:4184203), complete cds. [BC016934] |
| PTX3 | 13,8 | 13,1 | 9,2 | pentraxin 3, long (PTX3), mRNA [NM_002852] |
| SLC9A6 | 12,7 | 13,7 | 11,3 | solute carrier family 9 (sodium/hydrogen exchanger), member 6 (SLC9A6), transcript variant 1, mRNA [NM_001042537] |
| LMO2 | 11,6 | 12,4 | 4,6 | LIM domain only 2 (rhombotin-like 1) (LMO2), transcript variant 1, mRNA [NM_005574] |
| SDC4 | 11,4 | 7,7 | 2,6 | syndecan 4 (SDC4), mRNA [NM_002999] |
| RELB | 11,0 | 9,3 | 5,0 | v-rel reticuloendotheliosis viral oncogene homolog B (RELB), mRNA [NM_006509] |
| ELOVL7 | 10,8 | 10,3 | 5,0 | ELOVL fatty acid elongase 7 (ELOVL7), transcript variant 1, mRNA [NM_024930] |
| STATH | 10,7 | 13,2 | 10,9 | statherin (STATH), transcript variant 1, mRNA [NM_003154] |
| NCRNA00246A | 10,0 | 11,9 | 11,3 | non-protein coding RNA 246A (NCRNA00246A), non-coding RNA [NR_026595] |
| EREG | 9,2 | 13,8 | 4,1 | epiregulin (EREG), mRNA [NM_001432] |
| STX11 | 9,1 | 5,4 | 3,8 | syntaxin 11 (STX11), mRNA [NM_003764] |
| TNF | 8,7 | 2,5 | 2,8 | tumor necrosis factor (TNF), mRNA [NM_000594] |
| RAMP2 | 8,6 | 4,1 | 2,5 | receptor (G protein-coupled) activity modifying protein 2 (RAMP2), mRNA [NM_005854] |
| PTGS2 | 8,4 | 4,8 | 2,0 | prostaglandin-endoperoxide synthase 2 (prostaglandin G/H synthase and cyclooxygenase) (PTGS2), mRNA [NM_000963] |
| PP12613 | 7,5 | 9,3 | 7,3 | hypothetical LOC100192379 (PP12613), non-coding RNA [NR_024365] |
| CSF3 | 7,3 | 4,9 | 3,9 | colony stimulating factor 3 (granulocyte) (CSF3), transcript variant 1, mRNA [NM_000759] |
| ZC3H12A | 7,2 | 3,0 | 2,1 | zinc finger CCCH-type containing 12A (ZC3H12A), mRNA [NM_025079] |
| XAF1 | 7,0 | 5,0 | 3,7 | XIAP associated factor 1 (XAF1), transcript variant 1, mRNA [NM_017523] |
| LGALS9 | 6,6 | 7,6 | 7,8 | lectin, galactoside-binding, soluble, 9 (LGALS9), transcript variant 1, mRNA [NM_009587] |
| CYP4F11 | 6,5 | 8,0 | 6,5 | cytochrome P450, family 4, subfamily F, polypeptide 11 (CYP4F11), transcript variant 1, mRNA [NM_021187] |
| RSPH6A | 6,1 | 7,7 | 6,0 | radial spoke head 6 homolog A (Chlamydomonas) (RSPH6A), mRNA [NM_030785] |
| IER3 | 5,9 | 2,9 | 2,2 | immediate early response 3 (IER3), mRNA [NM_003897] |
| TMCC3 | 5,8 | 7,0 | 9,9 | transmembrane and coiled-coil domain family 3 (TMCC3), mRNA [NM_020698] |
| IL1B | 5,8 | 4,3 | 1,8 | interleukin 1, beta (IL1B), mRNA [NM_000576] |
| DUSP5 | 5,7 | 5,5 | 3,3 | dual specificity phosphatase 5 (DUSP5), mRNA [NM_004419] |
| P2RY8 | 5,7 | 4,7 | 4,7 | purinergic receptor P2Y, G-protein coupled, 8 (P2RY8), mRNA [NM_178129] |
| LOC100130691 | 5,5 | 6,0 | 5,7 | hypothetical LOC100130691 (LOC100130691), non-coding RNA [NR_026966] |
| HIST1H3I | 5,4 | 5,5 | 4,1 | histone cluster 1, H3i (HIST1H3I), mRNA [NM_003533] |
| PRSS36 | 5,3 | 5,7 | 5,3 | protease, serine, 36 (PRSS36), mRNA [NM_173502] |
| SOD2 | 5,2 | 6,5 | 5,8 | superoxide dismutase 2, mitochondrial (SOD2), nuclear gene encoding mitochondrial protein, mRNA [NM_001024465] |
| FBXW10 | 5,2 | 3,4 | 2,7 | F-box and WD repeat domain containing 10 (FBXW10), mRNA [NM_031456] |
| IL7 | 5,2 | 5,3 | 5,8 | interleukin 7 (IL7), transcript variant 1, mRNA [NM_000880] |
| CCL2 | 5,1 | 2,2 | -1,0 | chemokine (C-C motif) ligand 2 (CCL2), mRNA [NM_002982] |
| TACR1 | 5,1 | 7,6 | 8,5 | tachykinin receptor 1 (TACR1), transcript variant short, mRNA [NM_015727] |
| AREG | 5,1 | 2,9 | 1,8 | amphiregulin (AREG), mRNA [NM_001657] |
| PGBD5 | 5,0 | 4,2 | 6,4 | piggyBac transposable element derived 5 (PGBD5), mRNA [NM_024554] |
| SEMG2 | 5,0 | 6,7 | 8,3 | semenogelin II (SEMG2), mRNA [NM_003008] |
| MAFF | 5,0 | 4,0 | 1,9 | v-maf musculoaponeurotic fibrosarcoma oncogene homolog F (avian) (MAFF), transcript variant 1, mRNA [NM_012323] |
| CREB3L3 | 4,9 | 6,3 | 5,7 | cAMP responsive element binding protein 3-like 3 (CREB3L3), mRNA [NM_032607] |
| SAMD4A | 4,9 | 7,3 | 3,4 | sterile alpha motif domain containing 4A (SAMD4A), transcript variant 1, mRNA [NM_015589] |
| TMEM119 | 4,9 | 4,5 | 5,6 | transmembrane protein 119 (TMEM119), mRNA [NM_181724] |
| EGR1 | 4,7 | 3,6 | 1,4 | early growth response 1 (EGR1), mRNA [NM_001964] |
| FAM27L | 4,7 | 5,3 | 5,6 | family with sequence similarity 27-like (FAM27L), non-coding RNA [NR_028336] |
| RASD1 | 4,6 | 3,9 | 3,2 | RAS, dexamethasone-induced 1 (RASD1), transcript variant 1, mRNA [NM_016084] |
| SAT1 | 4,5 | 3,3 | 2,7 | spermidine/spermine N1-acetyltransferase 1 (SAT1), transcript variant 1, mRNA [NM_002970] |
| CYP3A5 | 4,5 | 6,1 | 5,7 | cytochrome P450, family 3, subfamily A, polypeptide 5 (CYP3A5), transcript variant 1, mRNA [NM_000777] |
| G0S2 | 4,3 | 3,0 | 1,3 | G0/G1switch 2 (G0S2), mRNA [NM_015714] |
| PHLDA1 | 4,3 | 3,5 | 2,4 | pleckstrin homology-like domain, family A, member 1 (PHLDA1), mRNA [NM_007350] |
| TNFSF14 | 4,3 | 2,0 | 3,0 | tumor necrosis factor (ligand) superfamily, member 14 (TNFSF14), transcript variant 1, mRNA [NM_003807] |
| CEACAM5 | 4,2 | 5,8 | 4,9 | carcinoembryonic antigen-related cell adhesion molecule 5 (CEACAM5), mRNA [NM_004363] |
| NFKBIZ | 4,2 | 1,2 | 1,8 | nuclear factor of kappa light polypeptide gene enhancer in B-cells inhibitor, zeta (NFKBIZ), mRNA [NM_031419] |
| LOC401317 | 4,2 | 4,5 | 1,2 | PREDICTED: hypothetical LOC401317 (LOC401317), partial miscRNA [XR_108761] |
| NFKBIA | 4,2 | 1,3 | 1,5 | nuclear factor of kappa light polypeptide gene enhancer in B-cells inhibitor, alpha (NFKBIA), mRNA [NM_020529] |
| JSRP1 | 4,1 | 3,8 | 7,3 | junctional sarcoplasmic reticulum protein 1 (JSRP1), mRNA [NM_144616] |
| TANC2 | 4,1 | 3,2 | 2,4 | cDNA FLJ10215 fis, clone HEMBA1006737. [AK001077] |
| B3GNT5 | 4,1 | 2,7 | 1,5 | UDP-GlcNAc:betaGal beta-1,3-N-acetylglucosaminyltransferase 5 (B3GNT5), mRNA [NM_032047] |
| NFKB2 | 4,1 | 3,7 | 2,2 | nuclear factor of kappa light polypeptide gene enhancer in B-cells 2 (p49/p100) (NFKB2), [NM_001077493] |
| ZNF135 | 4,1 | 4,6 | 4,5 | zinc finger protein 135 (ZNF135), transcript variant 2, mRNA [NM_003436] |
| PGF | 4,1 | 2,4 | 2,8 | placental growth factor (PGF), transcript variant 1, mRNA [NM_002632] |
| NFKB1 | 4,0 | 2,2 | 1,4 | nuclear factor of kappa light polypeptide gene enhancer in B-cells 1 (NFKB1), transcript variant 1, mRNA [NM_003998] |
| ZIM3 | 4,0 | 3,1 | 2,4 | zinc finger, imprinted 3 (ZIM3), mRNA [NM_052882] |
| LOC100505974 | 4,0 | 4,2 | 3,6 | PREDICTED: hypothetical LOC100505974 (LOC100505974), partial miscRNA [XR_109690] |
| GCH1 | 4,0 | 2,8 | 1,7 | GTP cyclohydrolase 1 (GCH1), transcript variant 1, mRNA [NM_000161] |
| RHO | 4,0 | 4,5 | 3,8 | rhodopsin (RHO), mRNA [NM_000539] |
| ZNF577 | 4,0 | 4,3 | 3,7 | zinc finger protein 577 (ZNF577), transcript variant 3, non-coding RNA [NR_024181] |
| PIK3IP1 | 4,0 | 7,0 | 6,0 | phosphoinositide-3-kinase interacting protein 1 (PIK3IP1), transcript variant 1, mRNA [NM_052880] |
| SULT2B1 | 4,0 | 5,7 | 4,7 | sulfotransferase family, cytosolic, 2B, member 1 (SULT2B1), transcript variant 1, mRNA [NM_004605] |
| PNCK | 4,0 | 6,3 | 6,2 | pregnancy up-regulated non-ubiquitously expressed CaM kinase (PNCK), transcript variant 1, mRNA [NM_001039582] |
| GHR | 4,0 | 5,3 | 4,9 | growth hormone receptor (GHR), transcript variant 1, mRNA [NM_000163] |
| NFATC1 | 4,0 | 1,1 | 1,1 | nuclear factor of activated T-cells, cytoplasmic, calcineurin-dependent 1 (NFATC1), transcript variant 3, mRNA [NM_172387] |
| PCDHB6 | 4,0 | 3,4 | 2,4 | protocadherin beta 6 (PCDHB6), mRNA [NM_018939] |
| BDKRB1 | 3,9 | 4,8 | 2,7 | bradykinin receptor B1 (BDKRB1), mRNA [NM_000710] |
| ADAMTS17 | 3,9 | 3,9 | 4,2 | ADAM metallopeptidase with thrombospondin type 1 motif, 17 (ADAMTS17), mRNA [NM_139057] |
| HRASLS5 | 3,8 | 4,2 | 3,5 | HRAS-like suppressor family, member 5 (HRASLS5), transcript variant 1, mRNA [NM_054108] |
| INHBA | 3,8 | 4,7 | 3,1 | inhibin, beta A (INHBA), mRNA [NM_002192] |
| LIF | 3,8 | 2,4 | 1,9 | leukemia inhibitory factor (cholinergic differentiation factor) (LIF), mRNA [NM_002309] |
| LOC100506983 | 3,8 | 6,2 | 5,1 | PREDICTED: hypothetical LOC100506983, transcript variant 1 (LOC100506983), partial miscRNA [XR_109216] |
| ZNF682 | 3,8 | 3,3 | 2,4 | zinc finger protein 682 (ZNF682), transcript variant 1, mRNA [NM_033196] |
| CST9L | 3,7 | 3,9 | 5,8 | cystatin 9-like (CST9L), mRNA [NM_080610] |
| SRG7 | 3,7 | 5,3 | 6,0 | spermatogenesis-related protein 7 (SRG7), non-coding RNA [NR_034168] |
| LOC646626 | 3,7 | 3,7 | 2,7 | PREDICTED: hypothetical protein LOC646626 (LOC646626), mRNA [XM_942822] |
| HIVEP3 | 3,7 | 3,8 | 2,3 | human immunodeficiency virus type I enhancer binding protein 3 [Source:HGNC Symbol |
| MOV10L1 | 3,7 | 4,6 | 3,3 | Mov10l1, Moloney leukemia virus 10-like 1, homolog (mouse) (MOV10L1), transcript variant 1, mRNA [NM_018995] |
| TEKT1 | 3,7 | 3,2 | 2,5 | tektin 1 (TEKT1), mRNA [NM_053285] |
| SCN4A | 3,7 | 5,5 | 4,9 | sodium channel, voltage-gated, type IV, alpha subunit (SCN4A), mRNA [NM_000334] |
| BTN3A1 | 3,6 | 6,4 | 4,4 | butyrophilin, subfamily 3, member A1 (BTN3A1), transcript variant 1, mRNA [NM_007048] |
| TNIP1 | 3,6 | 5,0 | 3,0 | TNFAIP3 interacting protein 1 (TNIP1), mRNA [NM_006058] |
| FAM131B | 3,6 | 6,2 | 4,9 | family with sequence similarity 131, member B (FAM131B), transcript variant b, mRNA [NM_014690] |
| PRO2949 | 3,6 | 5,7 | 6,9 | PRO2949 mRNA, complete cds. [AF119907] |
| DNAH5 | 3,6 | 4,8 | 4,9 | dynein, axonemal, heavy chain 5 (DNAH5), mRNA [NM_001369] |
| HEATR7B2 | 3,5 | 7,0 | 7,4 | HEAT repeat family member 7B2 (HEATR7B2), mRNA [NM_173489] |
| TRAF3IP3 | 3,5 | 2,8 | 2,3 | TRAF3 interacting protein 3 (TRAF3IP3), mRNA [NM_025228] |
| SLC30A4 | 3,5 | 5,0 | 3,7 | solute carrier family 30 (zinc transporter), member 4 [Source:HGNC Symbol |
| CRYBA2 | 3,5 | 3,7 | 2,8 | crystallin, beta A2 (CRYBA2), transcript variant 1, mRNA [NM_005209] |
| BEND7 | 3,5 | 6,5 | 8,9 | BEN domain containing 7 (BEND7), transcript variant 1, mRNA [NM_152751] |
| HBM | 3,4 | 3,7 | 3,4 | hemoglobin, mu (HBM), mRNA [NM_001003938] |
| KCNB2 | 3,4 | 3,1 | 2,4 | potassium voltage-gated channel, Shab-related subfamily, member 2 (KCNB2), mRNA [NM_004770] |
| NACC1 | 3,4 | 4,0 | 3,5 | nucleus accumbens associated 1, BEN and BTB (POZ) domain containing (NACC1), mRNA [NM_052876] |
| DDX58 | 3,4 | 2,7 | 1,1 | DEAD (Asp-Glu-Ala-Asp) box polypeptide 58 (DDX58), mRNA [NM_014314] |
| TNFAIP2 | 3,4 | 2,2 | 1,1 | tumor necrosis factor, alpha-induced protein 2 (TNFAIP2), mRNA [NM_006291] |
| ZC3H12C | 3,4 | 2,0 | 1,0 | zinc finger CCCH-type containing 12C (ZC3H12C), mRNA [NM_033390] |
| TMEM201 | 3,3 | 3,7 | 3,2 | transmembrane protein 201 (TMEM201), transcript variant 1, mRNA [NM_001130924] |
| TNFAIP3 | 3,3 | 1,9 | 1,5 | tumor necrosis factor, alpha-induced protein 3 (TNFAIP3), mRNA [NM_006290] |
| IL11 | 3,3 | 3,1 | 2,5 | interleukin 11 (IL11), mRNA [NM_000641] |
| SAA2 | 3,3 | 8,6 | 4,6 | cDNA clone IMAGE:4716555, with apparent retained intron. [BC058008] |
| PIH2 | 3,3 | 3,4 | 2,5 | pregnancy-induced hypertension syndrome-related protein (PIH2) mRNA, partial cds. [AF232217] |
| GLTSCR1 | 3,3 | 3,8 | 3,4 | glioma tumor suppressor candidate region protein 1 (GLTSCR1) mRNA, complete cds. [AF182077] |
| GATM | 3,3 | 3,0 | 1,0 | glycine amidinotransferase (L-arginine:glycine amidinotransferase) (GATM), mRNA [NM_001482] |
| NFRKB | 3,3 | 3,9 | 2,3 | nuclear factor related to kappaB binding protein (NFRKB), transcript variant 2, mRNA [NM_006165] |
| MAFIP | 3,3 | 4,3 | 3,0 | cDNA FLJ39633 fis, clone SMINT2002457, weakly similar to Tektin A1. [AK096952] |
| GYPE | 3,3 | 2,5 | 1,9 | glycophorin E (MNS blood group) (GYPE), transcript variant 1, mRNA [NM_002102] |
| SPP1 | 3,3 | 3,9 | 3,9 | secreted phosphoprotein 1 (SPP1), transcript variant 1, mRNA [NM_001040058] |
| FOSL1 | 3,3 | 4,5 | 2,8 | FOS-like antigen 1 (FOSL1), mRNA [NM_005438] |
| CEBPD | 3,2 | 1,5 | 1,1 | CCAAT/enhancer binding protein (C/EBP), delta (CEBPD), mRNA [NM_005195] |
| SRXN1 | 3,2 | 4,0 | 2,1 | sulfiredoxin 1 (SRXN1), mRNA [NM_080725] |
| KBTBD8 | 3,2 | 2,5 | 3,3 | kelch repeat and BTB (POZ) domain containing 8 (KBTBD8), mRNA [NM_032505] |
| NLN | 3,2 | 3,0 | 2,2 | neurolysin (metallopeptidase M3 family) [Source:HGNC Symbol |
| WFDC10A | 3,2 | 10,0 | 10,1 | WAP four-disulfide core domain 10A (WFDC10A), mRNA [NM_080753] |
| NFKBIE | 3,2 | 1,7 | 1,8 | nuclear factor of kappa light polypeptide gene enhancer in B-cells inhibitor, epsilon (NFKBIE), mRNA [NM_004556] |
| WTAP | 3,2 | 1,2 | -1,1 | Wilms tumor 1 associated protein (WTAP), transcript variant 1, mRNA [NM_004906] |
| ZNF697 | 3,2 | 4,2 | 5,2 | zinc finger protein 697 (ZNF697), mRNA [NM_001080470] |
| RECQL | 3,2 | 3,3 | 2,5 | RecQ protein-like (DNA helicase Q1-like) (RECQL), transcript variant 1, mRNA [NM_002907] |
| BIRC2 | 3,2 | 2,5 | 1,4 | baculoviral IAP repeat containing 2 (BIRC2), mRNA [NM_001166] |
| PAQR6 | 3,2 | 1,2 | 1,0 | progestin and adipoQ receptor family member VI (PAQR6), transcript variant 1, mRNA [NM_024897] |
| AGBL4 | 3,1 | 3,3 | 2,6 | ATP/GTP binding protein-like 4 [Source:HGNC Symbol |
| LRRC4C | 3,1 | 3,0 | 8,0 | leucine rich repeat containing 4C (LRRC4C), mRNA [NM_020929] |
| RNF148 | 3,1 | 4,6 | 6,8 | ring finger protein 148 (RNF148), mRNA [NM_198085] |
| SERPINB8 | 3,1 | 3,5 | 2,7 | serpin peptidase inhibitor, clade B (ovalbumin), member 8 (SERPINB8), transcript variant 3, mRNA [NM_001031848] |
| RSPH9 | 3,1 | 3,7 | 3,6 | radial spoke head 9 homolog (Chlamydomonas) [Source:HGNC Symbol |
| NCOA7 | 3,1 | 2,4 | 1,5 | nuclear receptor coactivator 7 (NCOA7), transcript variant 1, mRNA [NM_181782] |
| DNHD1 | 3,1 | 2,3 | 1,6 | dynein heavy chain domain 1 (DNHD1), transcript variant 2, mRNA [NM_173589] |
| GFPT2 | 3,1 | 1,9 | 1,2 | glutamine-fructose-6-phosphate transaminase 2 (GFPT2), mRNA [NM_005110] |
| MX1 | 3,1 | 3,3 | 3,9 | myxovirus (influenza virus) resistance 1, interferon-inducible protein p78 (mouse) (MX1), , mRNA [NM_002462] |
| HHIP | 3,1 | 2,7 | 2,1 | hedgehog interacting protein [Source:HGNC Symbol |
| ANKRD55 | 3,1 | 4,8 | 3,1 | ankyrin repeat domain 55 (ANKRD55), mRNA [NM_024669] |
| SLFN5 | 3,1 | 1,4 | 1,5 | schlafen family member 5 (SLFN5), mRNA [NM_144975] |
| EID3 | 3,0 | 3,2 | 1,9 | EP300 interacting inhibitor of differentiation 3 (EID3), mRNA [NM_001008394] |
| UNC80 | 3,0 | 4,3 | 6,4 | unc-80 homolog (C. elegans) [Source:HGNC Symbol |
| CLDN1 | 3,0 | 4,6 | 3,3 | claudin 1 (CLDN1), mRNA [NM_021101] |
| DUSP6 | 3,0 | 2,7 | 2,7 | dual specificity phosphatase 6 (DUSP6), transcript variant 1, mRNA [NM_001946] |
| SMOX | 3,0 | 2,2 | 1,3 | spermine oxidase (SMOX), transcript variant 1, mRNA [NM_175839] |
| PLA2G7 | 3,0 | 9,9 | 3,0 | phospholipase A2, group VII (platelet-activating factor acetylhydrolase, plasma) (PLA2G7), , mRNA [NM_005084] |
| LOC387895 | 3,0 | 4,2 | 3,7 | hypothetical LOC387895 (LOC387895), non-coding RNA [NR_033878] |
| ARHGEF15 | 3,0 | 3,4 | 2,9 | Rho guanine nucleotide exchange factor (GEF) 15 (ARHGEF15), transcript variant 1, mRNA [NM_173728] |
| GBP3 | 3,0 | 3,0 | 1,7 | guanylate binding protein 3 (GBP3), mRNA [NM_018284] |
| MYH8 | 3,0 | 3,7 | 3,2 | myosin, heavy chain 8, skeletal muscle, perinatal (MYH8), mRNA [NM_002472] |
| TNFAIP6 | 3,0 | 2,4 | 2,2 | tumor necrosis factor, alpha-induced protein 6 (TNFAIP6), mRNA [NM_007115] |
| GIPC2 | 3,0 | 4,0 | 6,5 | GIPC PDZ domain containing family, member 2 (GIPC2), mRNA [NM_017655] |
| TMEM200A | 3,0 | 4,9 | 2,7 | transmembrane protein 200A, mRNA (cDNA clone MGC:50847 IMAGE:5760073), complete cds. [BC044246] |
| CFB | 3,0 | 2,5 | 2,5 | complement factor B (CFB), mRNA [NM_001710] |
| SYNE1 | 3,0 | 4,1 | 4,3 | mRNA for KIAA1756 protein, partial cds. [AB051543] |
| MT1B | 3,0 | 4,0 | 3,0 | metallothionein 1B (MT1B), mRNA [NM_005947] |
| CREB5 | 3,0 | 4,3 | 2,3 | cAMP responsive element binding protein 5 [Source:HGNC Symbol |
| EFNA1 | 3,0 | 1,1 | -1,0 | ephrin-A1 (EFNA1), transcript variant 1, mRNA [NM_004428] |
| LCN2 | 3,0 | 5,7 | 5,0 | lipocalin 2 (LCN2), mRNA [NM_005564] |
| RSAD2 | 3,0 | 3,7 | 2,1 | radical S-adenosyl methionine domain containing 2 (RSAD2), mRNA [NM_080657] |
| CPA2 | 3,0 | 4,3 | 2,9 | carboxypeptidase A2 (pancreatic) (CPA2), mRNA [NM_001869] |
| SERTAD1 | 3,0 | 1,3 | 1,2 | SERTA domain containing 1 (SERTAD1), mRNA [NM_013376] |
| SHROOM2 | 3,0 | 3,5 | 2,4 | shroom family member 2 (SHROOM2), mRNA [NM_001649] |
| ABTB2 | 3,0 | 1,8 | 1,3 | ankyrin repeat and BTB (POZ) domain containing 2 (ABTB2), mRNA [NM_145804] |
| GRK4 | 3,0 | 1,9 | 2,8 | G protein-coupled receptor kinase 4 (GRK4), transcript variant 3, mRNA [NM_001004057] |
| TMX4 | 3,0 | 3,9 | 4,4 | thioredoxin-related transmembrane protein 4 [Source:HGNC Symbol |
| EIF4E | 2,9 | 4,4 | 5,2 | eukaryotic translation initiation factor 4E (EIF4E), transcript variant 1, mRNA [NM_001968] |
| GATS | 2,9 | 2,9 | 3,1 | GATS, stromal antigen 3 opposite strand (GATS), transcript variant 1, mRNA [NM_178831] |
| NINJ1 | 2,9 | 2,6 | 1,5 | ninjurin 1 (NINJ1), mRNA [NM_004148] |
| LIX1 | 2,9 | 3,6 | 4,3 | Lix1 homolog (chicken) (LIX1), mRNA [NM_153234] |
| TIFA | 2,9 | 1,3 | 1,0 | TRAF-interacting protein with forkhead-associated domain (TIFA), mRNA [NM_052864] |
| ANKRD33B | 2,9 | 2,2 | 2,0 | ankyrin repeat domain 33B (ANKRD33B), mRNA [NM_001164440] |
| PARP14 | 2,9 | 2,4 | 2,1 | poly (ADP-ribose) polymerase family, member 14 (PARP14), mRNA [NM_017554] |
| TCF15 | 2,9 | 3,3 | 3,0 | transcription factor 15 (basic helix-loop-helix) (TCF15), mRNA [NM_004609] |
| SULT2A1 | 2,9 | 3,4 | 2,6 | sulfotransferase family, cytosolic, 2A, dehydroepiandrosterone (DHEA)-preferring, member 1 (SULT2A1), mRNA [NM_003167] |
| DMRT2 | 2,9 | 3,6 | 6,5 | doublesex and mab-3 related transcription factor 2 (DMRT2), transcript variant 1, mRNA [NM_006557] |
| MT1X | 2,9 | 3,9 | 3,1 | metallothionein 1X (MT1X), mRNA [NM_005952] |
| DLGAP1 | 2,9 | 3,4 | 3,7 | discs, large (Drosophila) homolog-associated protein 1 (DLGAP1), transcript variant 1, mRNA [NM_004746] |
| PCSK6 | 2,9 | 4,2 | 3,9 | proprotein convertase subtilisin/kexin type 6 (PCSK6), transcript variant 3, mRNA [NM_138322] |
| NAMPT | 2,9 | 3,0 | 1,2 | nicotinamide phosphoribosyltransferase (NAMPT), mRNA [NM_005746] |
| PALM2-AKAP2 | 2,9 | 3,3 | 2,9 | PALM2-AKAP2 readthrough (PALM2-AKAP2), transcript variant 2, mRNA [NM_147150] |
| IER5 | 2,9 | 1,7 | 1,2 | immediate early response 5 (IER5), mRNA [NM_016545] |
| PNMAL1 | 2,9 | 3,5 | 2,6 | PNMA-like 1 (PNMAL1), transcript variant 1, mRNA [NM_018215] |
| IVL | 2,9 | 3,7 | 5,5 | involucrin (IVL), mRNA [NM_005547] |
| MGC42157 | 2,9 | 3,3 | 2,8 | cDNA clone IMAGE:4799398. [BC030111] |
| SLED1 | 2,9 | 3,0 | 2,3 | proteoglycan 3 pseudogene (SLED1), non-coding RNA [NR_003542] |
| CFHR2 | 2,9 | 4,3 | 2,8 | complement factor H-related 2 (CFHR2), mRNA [NM_005666] |
| CDC42EP2 | 2,8 | 3,5 | 1,7 | CDC42 effector protein (Rho GTPase binding) 2 (CDC42EP2), mRNA [NM_006779] |
| BLNK | 2,8 | 3,1 | 2,8 | B-cell linker (BLNK), transcript variant 1, mRNA [NM_013314] |
| TNR | 2,8 | 3,1 | 2,8 | tenascin R (restrictin, janusin) (TNR), mRNA [NM_003285] |
| GBX1 | 2,8 | 4,7 | 6,5 | gastrulation brain homeobox 1 (GBX1), mRNA [NM_001098834] |
| ACSM5 | 2,8 | 3,1 | 2,3 | acyl-CoA synthetase medium-chain family member 5 [Source:HGNC Symbol |
| KBTBD3 | 2,8 | 3,5 | 2,5 | kelch repeat and BTB (POZ) domain containing 3 (KBTBD3), transcript variant 2, mRNA [NM_198439] |
| DRD3 | 2,8 | 3,0 | 2,8 | dopamine receptor D3 (DRD3), transcript variant e, mRNA [NM_033663] |
| LOC643733 | 2,8 | 3,5 | 2,5 | caspase 4, apoptosis-related cysteine peptidase pseudogene (LOC643733), non-coding RNA [NR_034078] |
| RNF19B | 2,8 | 3,2 | 2,0 | ring finger protein 19B (RNF19B), transcript variant 1, mRNA [NM_153341] |
| CCL3 | 2,8 | 2,5 | 2,5 | mRNA for pLD78 peptide, complete cds. [D00044] |
| MAP2K3 | 2,8 | 2,3 | 1,4 | mitogen-activated protein kinase kinase 3 (MAP2K3), transcript variant B, mRNA [NM_145109] |
| IFIH1 | 2,8 | 1,8 | 1,1 | interferon induced with helicase C domain 1 (IFIH1), mRNA [NM_022168] |
| SPTBN4 | 2,8 | 3,1 | 2,1 | spectrin, beta, non-erythrocytic 4 (SPTBN4), transcript variant sigma1, mRNA [NM_020971] |
| CCKBR | 2,8 | 3,5 | 2,9 | cholecystokinin B receptor (CCKBR), mRNA [NM_176875] |
| HBEGF | 2,8 | 1,5 | 1,2 | heparin-binding EGF-like growth factor (HBEGF), mRNA [NM_001945] |
| SLC8A2 | 2,8 | 5,0 | 4,2 | solute carrier family 8 (sodium/calcium exchanger), member 2 (SLC8A2), mRNA [NM_015063] |
| PMAIP1 | 2,8 | 1,5 | 1,1 | phorbol-12-myristate-13-acetate-induced protein 1 (PMAIP1), mRNA [NM_021127] |
| LOC644192 | 2,8 | 4,7 | 3,7 | PREDICTED: hypothetical LOC644192 (LOC644192), partial miscRNA [XR_109225] |
| CDHR3 | 2,8 | 3,7 | 2,9 | cadherin-related family member 3 (CDHR3), mRNA [NM_152750] |
| PI3 | 2,8 | 4,6 | 3,5 | peptidase inhibitor 3, skin-derived (PI3), mRNA [NM_002638] |
| MT1G | 2,8 | 4,1 | 2,9 | metallothionein 1G (MT1G), mRNA [NM_005950] |
| TPPP3 | 2,8 | 4,6 | 3,9 | tubulin polymerization-promoting protein family member 3 (TPPP3), mRNA [NM_016140] |
| CNTNAP2 | 2,7 | 4,9 | 8,4 | contactin associated protein-like 2 (CNTNAP2), mRNA [NM_014141] |
| SYCE2 | 2,7 | 3,2 | 2,6 | synaptonemal complex central element protein 2 (SYCE2), mRNA [NM_001105578] |
| FGF17 | 2,7 | 5,4 | 4,5 | fibroblast growth factor 17 (FGF17), mRNA [NM_003867] |
| CADM3 | 2,7 | 3,8 | 3,1 | cell adhesion molecule 3 (CADM3), transcript variant 1, mRNA [NM_021189] |
| MYCT1 | 2,7 | 4,0 | 3,2 | myc target 1 (MYCT1), mRNA [NM_025107] |
| ZDHHC11 | 2,7 | 1,4 | 1,2 | zinc finger, DHHC-type containing 11 (ZDHHC11), mRNA [NM_024786] |
| MAP3K8 | 2,7 | 1,7 | 1,6 | mitogen-activated protein kinase kinase kinase 8 (MAP3K8), mRNA [NM_005204] |
| PSG9 | 2,7 | 7,5 | 7,3 | pregnancy specific beta-1-glycoprotein 9 (PSG9), mRNA [NM_002784] |
| LOC100507203 | 2,7 | 3,6 | 3,6 | hypothetical LOC285733 (LOC285733), mRNA [NM_001195597] |
| NPSA | 2,7 | 5,2 | 4,8 | prostate-specific antigen mRNA, complete cds. [AF527974] |
| ADH6 | 2,7 | 3,6 | 2,7 | alcohol dehydrogenase 6 (class V) (ADH6), transcript variant 1, mRNA [NM_001102470] |
| LOC100506123 | 2,7 | -1,0 | -1,4 | hypothetical LOC100506123 (LOC100506123), non-coding RNA [NR_040097] |
| ARHGAP20 | 2,7 | 7,6 | 5,5 | Rho GTPase activating protein 20 (ARHGAP20), mRNA [NM_020809] |
| LILRB5 | 2,7 | 6,2 | 6,2 | leukocyte immunoglobulin-like receptor, subfamily B , member 5 (LILRB5), transcript variant 2, mRNA [NM_006840] |
| PLA2G2F | 2,7 | 3,8 | 3,2 | phospholipase A2, group IIF (PLA2G2F), mRNA [NM_022819] |
| ICAM1 | 2,7 | 3,1 | 2,5 | intercellular adhesion molecule 1 (ICAM1), mRNA [NM_000201] |
| FGF16 | 2,7 | 3,4 | 3,0 | fibroblast growth factor 16 (FGF16), mRNA [NM_003868] |
| SEMA7A | 2,7 | 4,1 | 4,5 | semaphorin 7A, GPI membrane anchor (John Milton Hagen blood group) (SEMA7A), transcript variant 1, mRNA [NM_003612] |
| FLG | 2,7 | 3,5 | 2,6 | filaggrin (FLG), mRNA [NM_002016] |
| LOC100506546 | 2,7 | 4,2 | 4,1 | PREDICTED: hypothetical LOC100506546, transcript variant 2 (LOC100506546), partial miscRNA [XR_114959] |
| USP9Y | 2,7 | 3,5 | 2,5 | ubiquitin specific peptidase 9, Y-linked (USP9Y), mRNA [NM_004654] |
| MT1L | 2,7 | 3,4 | 2,7 | metallothionein 1L (gene/pseudogene) (MT1L), non-coding RNA [NR_001447] |
| PANX1 | 2,6 | 3,5 | 2,5 | pannexin 1 (PANX1), mRNA [NM_015368] |
| IFNA16 | 2,6 | 3,6 | 4,4 | interferon, alpha 16 (IFNA16), mRNA [NM_002173] |
| EHF | 2,6 | 2,7 | 3,1 | ets homologous factor (EHF), transcript variant 1, mRNA [NM_001206616] |
| CNKSR1 | 2,6 | 4,5 | 4,0 | connector enhancer of kinase suppressor of Ras 1 (CNKSR1), transcript variant 1, mRNA [NM_006314] |
| GLS | 2,6 | 2,1 | 1,8 | glutaminase (GLS), nuclear gene encoding mitochondrial protein, mRNA [NM_014905] |
| IGLL1 | 2,6 | 3,2 | 2,6 | immunoglobulin lambda-like polypeptide 1 (IGLL1), transcript variant 1, mRNA [NM_020070] |
| IL26 | 2,6 | 3,4 | 2,9 | interleukin 26 (IL26), mRNA [NM_018402] |
| SLC26A10 | 2,6 | 3,5 | 2,8 | solute carrier family 26, member 10 (SLC26A10), mRNA [NM_133489] |
| DNAJC6 | 2,6 | 3,6 | 4,3 | DnaJ (Hsp40) homolog, subfamily C, member 6 (DNAJC6), mRNA [NM_014787] |
| POM121L8P | 2,6 | 4,7 | 4,2 | POM121 membrane glycoprotein-like 8 pseudogene (POM121L8P), non-coding RNA [NR_024583] |
| SMAD3 | 2,6 | 3,7 | 1,7 | mad protein homolog (hMAD-3) mRNA, complete cds. [U68019] |
| CBR3 | 2,6 | 2,8 | 1,5 | carbonyl reductase 3 (CBR3), mRNA [NM_001236] |
| ABCC8 | 2,6 | 3,5 | 3,2 | ATP-binding cassette, sub-family C (CFTR/MRP), member 8 (ABCC8), mRNA [NM_000352] |
| SLC12A7 | 2,6 | 2,4 | 1,5 | solute carrier family 12 (potassium/chloride transporters), member 7 (SLC12A7), mRNA [NM_006598] |
| HAVCR2 | 2,6 | 3,6 | 2,8 | hepatitis A virus cellular receptor 2 (HAVCR2), mRNA [NM_032782] |
| FSD2 | 2,6 | 4,1 | 3,1 | mRNA |
| LOC100507419 | 2,6 | 2,6 | 3,0 | PREDICTED: hypothetical protein LOC100507419 (LOC100507419), mRNA [XM_003118684] |
| ABCA5 | 2,6 | 2,8 | 2,3 | ATP-binding cassette, sub-family A (ABC1), member 5 (ABCA5), transcript variant 1, mRNA [NM_018672] |
| TMEM8A | 2,6 | 3,9 | 3,2 | transmembrane protein 8A [Source:HGNC Symbol |
| LRRC2 | 2,6 | 3,7 | 2,5 | leucine rich repeat containing 2 (LRRC2), mRNA [NM_024512] |
| SCML2 | 2,6 | 3,6 | 2,5 | sex comb on midleg-like 2 (Drosophila) (SCML2), transcript variant 1, mRNA [NM_006089] |
| FRMPD4 | 2,6 | 3,4 | 3,2 | FERM and PDZ domain containing 4 (FRMPD4), mRNA [NM_014728] |
| KLF14 | 2,6 | 3,2 | 2,2 | Kruppel-like factor 14 (KLF14), mRNA [NM_138693] |
| ZNF347 | 2,6 | 5,8 | 3,9 | zinc finger protein 347 (ZNF347), transcript variant 3, mRNA [NM_032584] |
| TAS2R20 | 2,6 | 3,6 | 2,5 | taste receptor, type 2, member 20 (TAS2R20), mRNA [NM_176889] |
| WDR49 | 2,6 | 7,1 | 7,7 | WD repeat domain 49 (WDR49), mRNA [NM_178824] |
| SP100 | 2,6 | 2,0 | 1,8 | nuclear autoantigen mRNA, partial cds |
| INSM2 | 2,6 | 3,6 | 2,5 | insulinoma-associated 2 (INSM2), mRNA [NM_032594] |
| EPB41L5 | 2,6 | 3,8 | 4,9 | erythrocyte membrane protein band 4.1 like 5 (EPB41L5), transcript variant 1, mRNA [NM_020909] |
| FAM19A1 | 2,6 | 3,6 | 2,5 | family with sequence similarity 19 (chemokine (C-C motif)-like), member A1 (FAM19A1), mRNA [NM_213609] |
| CASQ2 | 2,6 | 3,6 | 2,5 | calsequestrin 2 (cardiac muscle) (CASQ2), nuclear gene encoding mitochondrial protein, mRNA [NM_001232] |
| GUCY1A3 | 2,6 | 3,6 | 2,5 | guanylate cyclase 1, soluble, alpha 3 (GUCY1A3), transcript variant 3, mRNA [NM_001130683] |
| TAPT1 | 2,6 | 3,6 | 2,5 | transmembrane anterior posterior transformation 1 [Source:HGNC Symbol |
| KCNJ14 | 2,6 | 1,2 | 1,8 | potassium inwardly-rectifying channel, subfamily J, member 14 (KCNJ14), transcript variant 2, mRNA [NM_170720] |
| LOC100507033 | 2,6 | 3,1 | 2,4 | PREDICTED: hypothetical LOC100507033 (LOC100507033), partial miscRNA [XR_110380] |
| FNDC7 | 2,6 | 3,6 | 2,5 | fibronectin type III domain containing 7 (FNDC7), mRNA [NM_001144937] |
| ESAM | 2,6 | 3,6 | 2,5 | endothelial cell adhesion molecule (ESAM), mRNA [NM_138961] |
| KLRF1 | 2,6 | 3,3 | 2,6 | killer cell lectin-like receptor subfamily F, member 1 (KLRF1), mRNA [NM_016523] |
| ZNF333 | 2,6 | 3,2 | 2,4 | zinc finger protein 333 [Source:HGNC Symbol |
| TSPYL5 | 2,6 | 3,6 | 2,7 | TSPY-like 5 (TSPYL5), mRNA [NM_033512] |
| CTSS | 2,6 | 3,5 | 2,5 | cathepsin S (CTSS), transcript variant 1, mRNA [NM_004079] |
| LOC441495 | 2,6 | 3,4 | 5,8 | centromere protein V pseudogene (LOC441495), non-coding RNA [NR_033773] |
| RBM46 | 2,6 | 3,5 | 2,6 | RNA binding motif protein 46 (RBM46), mRNA [NM_144979] |
| LRRC27 | 2,6 | 3,4 | 2,6 | leucine rich repeat containing 27 (LRRC27), transcript variant 1, mRNA [NM_030626] |
| PDZD9 | 2,6 | 3,5 | 3,9 | PDZ domain containing 9 (PDZD9), transcript variant 1, mRNA [NM_173806] |
| IQCF1 | 2,6 | 3,4 | 2,6 | IQ motif containing F1, mRNA (cDNA clone IMAGE:5266078), complete cds. [BC034228] |
| PCDH15 | 2,6 | 3,4 | 2,6 | mRNA |
| KCNE2 | 2,6 | 5,7 | 7,0 | potassium voltage-gated channel, Isk-related family, member 2 (KCNE2), mRNA [NM_172201] |
| AKT3 | 2,6 | 3,4 | 2,5 | v-akt murine thymoma viral oncogene homolog 3 (protein kinase B, gamma) (AKT3), transcript variant 1, mRNA [NM_005465] |
| ERBB2 | 2,6 | 3,5 | 2,4 | v-erb-b2 erythroblastic leukemia viral oncogene homolog 2, neuro/glioblastoma derived oncogene homolog (avian |
| TPD52L3 | 2,6 | 3,5 | 3,2 | tumor protein D52-like 3 (TPD52L3), transcript variant 1, mRNA [NM_033516] |
| SPEM1 | 2,6 | 6,1 | 7,2 | spermatid maturation 1 (SPEM1), mRNA [NM_199339] |
| WNT10A | 2,6 | 4,0 | 3,4 | wingless-type MMTV integration site family, member 10A (WNT10A), mRNA [NM_025216] |
| RNF8 | 2,6 | 3,5 | 2,6 | ring finger protein 8 (RNF8), transcript variant 1, mRNA [NM_003958] |
|  |  |  |  |  |
| **Gene symbol** | **t_0_** | **t_3_** | **t_6_** | **Description** |
| **Downregulated genes** | | | | |
| CDC42BPG | -2,4 | -7,4 | -1,7 | CDC42 binding protein kinase gamma (DMPK-like) [Source:HGNC Symbol |
| METTL7A | -4,0 | -6,6 | -4,4 | methyltransferase like 7A (METTL7A), mRNA [NM_014033] |
| LOC643401 | -3,0 | -6,6 | -2,8 | hypothetical protein LOC340109, mRNA (cDNA clone IMAGE:5578073), partial cds. [BC039509] |
| NFE2 | -9,0 | -5,5 | -1,7 | nuclear factor (erythroid-derived 2), 45kDa (NFE2), transcript variant 1, mRNA [NM_006163] |
| SLC24A3 | -2,1 | -5,4 | -2,8 | solute carrier family 24 (sodium/potassium/calcium exchanger), member 3 (SLC24A3), mRNA [NM_020689] |
| NKX3-2 | -7,3 | -5,3 | -2,4 | NK3 homeobox 2 (NKX3-2), mRNA [NM_001189] |
| CXXC4 | -9,7 | -5,2 | -1,5 | CXXC finger protein 4 (CXXC4), mRNA [NM_025212] |
| TRIM66 | -1,6 | -5,1 | -1,3 | tripartite motif containing 66 (TRIM66), mRNA [NM_014818] |
| CNR1 | -2,8 | -5,1 | -2,1 | cannabinoid receptor 1 (brain) (CNR1), transcript variant 2, mRNA [NM_033181] |
| ARMC9 | -1,5 | -5,0 | -1,1 | armadillo repeat containing 9 [Source:HGNC Symbol |
| BNC2 | -1,7 | -4,8 | -1,4 | basonuclin 2 (BNC2), mRNA [NM_017637] |
| TRIM6 | -4,5 | -4,7 | -1,7 | tripartite motif containing 6 (TRIM6), transcript variant 1, mRNA [NM_001003818] |
| CEBPA | -3,5 | -4,6 | -7,4 | CCAAT/enhancer binding protein (C/EBP), alpha (CEBPA), mRNA [NM_004364] |
| PLCXD3 | -4,2 | -4,4 | -1,9 | phosphatidylinositol-specific phospholipase C, X domain containing 3 (PLCXD3), mRNA [NM_001005473] |
| PCBD2 | -2,2 | -4,3 | -1,9 | pterin-4 alpha-carbinolamine dehydratase/dimerization cofactor of hepatocyte nuclear factor 1 alpha (TCF1) 2 [Source:HGNC Symbol |
| NUDT18 | -2,6 | -4,3 | 1,1 | nudix (nucleoside diphosphate linked moiety X)-type motif 18 (NUDT18), mRNA [NM_024815] |
| PTPLAD2 | -1,7 | -4,2 | -1,2 | protein tyrosine phosphatase-like A domain containing 2 (PTPLAD2), mRNA [NM_001010915] |
| RFX3 | -1,5 | -4,1 | -1,4 | regulatory factor X, 3 (influences HLA class II expression) [Source:HGNC Symbol |
| AGBL1 | -3,1 | -4,1 | -1,8 | cDNA FLJ32310 fis, clone PROST2003102. [AK056872] |
| PRKCB | -2,6 | -4,0 | -2,5 | protein kinase C, beta (PRKCB), transcript variant 2, mRNA [NM_002738] |
| COX18 | -3,0 | -3,9 | -4,5 | COX18 cytochrome c oxidase assembly homolog (S. cerevisiae) (COX18), nuclear gene encoding mitochondrial protein, mRNA [NM_173827] |
| LOC100505956 | -2,3 | -3,9 | -1,2 | PREDICTED: hypothetical LOC100505956 (LOC100505956), partial miscRNA [XR_110884] |
| RBM14 | 1,1 | -3,9 | -1,8 | RNA binding motif protein 14 (RBM14), transcript variant 1, mRNA [NM_006328] |
| KRT19 | -2,5 | -3,9 | -1,9 | keratin 19 (KRT19), mRNA [NM_002276] |
| WDR60 | -1,9 | -3,8 | -2,1 | WD repeat domain 60 (WDR60), mRNA [NM_018051] |
| PDE8B | -2,4 | -3,8 | -1,6 | phosphodiesterase 8B (PDE8B), transcript variant 1, mRNA [NM_003719] |
| CEP290 | -1,8 | -3,8 | -4,0 | centrosomal protein 290kDa (CEP290), mRNA [NM_025114] |
| DLC1 | -3,4 | -3,8 | -1,8 | deleted in liver cancer 1 (DLC1), transcript variant 1, mRNA [NM_182643] |
| RBM43 | -2,0 | -3,8 | -1,4 | RNA binding motif protein 43 (RBM43), mRNA [NM_198557] |
| LRRIQ1 | -1,6 | -3,8 | -2,5 | leucine-rich repeats and IQ motif containing 1 (LRRIQ1), mRNA [NM_001079910] |
| MAP1A | -1,4 | -3,7 | -1,2 | microtubule-associated protein 1A (MAP1A), mRNA [NM_002373] |
| GABPB2 | 1,2 | -3,7 | -2,1 | GA binding protein transcription factor, beta subunit 2 [Source:HGNC Symbol |
| TRERF1 | -4,9 | -3,7 | -3,9 | transcriptional regulating factor 1 (TRERF1), mRNA [NM_033502] |
| RAB3C | -1,4 | -3,7 | -3,2 | RAB3C, member RAS oncogene family [Source:HGNC Symbol |
| C1QTNF3 | -2,4 | -3,6 | -1,8 | C1q and tumor necrosis factor related protein 3 (C1QTNF3), transcript variant 2, mRNA [NM_181435] |
| DNAJC21 | -1,3 | -3,6 | -2,9 | DnaJ (Hsp40) homolog, subfamily C, member 21 (DNAJC21), transcript variant 1, mRNA [NM_194283] |
| NYNRIN | -2,4 | -3,5 | -1,2 | NYN domain and retroviral integrase containing (NYNRIN), mRNA [NM_025081] |
| ACAD11 | -1,3 | -3,5 | -2,3 | acyl-CoA dehydrogenase family, member 11 (ACAD11), mRNA [NM_032169] |
| NCKAP5 | -1,5 | -3,5 | -1,9 | NCK-associated protein 5 (NCKAP5), transcript variant 1, mRNA [NM_207363] |
| FUBP1 | -3,1 | -3,5 | -4,3 | far upstream element (FUSE) binding protein 1 [Source:HGNC Symbol |
| ARID5B | -3,9 | -3,5 | -1,4 | AT rich interactive domain 5B (MRF1-like) (ARID5B), mRNA [NM_032199] |
| PCDH11Y | -3,3 | -3,5 | -3,0 | protocadherin 11 Y-linked (PCDH11Y), transcript variant c, mRNA [NM_032973] |
| CA9 | 1,0 | -3,5 | -5,5 | carbonic anhydrase IX (CA9), mRNA [NM_001216] |
| NCRNA00174 | -1,4 | -3,5 | -1,2 | non-protein coding RNA 174 (NCRNA00174), non-coding RNA [NR_026873] |
| DHDDS | -1,2 | -3,5 | -1,4 | dehydrodolichyl diphosphate synthase (DHDDS), transcript variant 1, mRNA [NM_024887] |
| SIRT3 | -2,1 | -3,4 | -1,3 | sirtuin 3 (SIRT3), nuclear gene encoding mitochondrial protein, transcript variant 1, mRNA [NM_012239] |
| KRCC1 | 1,1 | -3,4 | 1,3 | lysine-rich coiled-coil 1 (KRCC1), mRNA [NM_016618] |
| ABCA11P | -1,3 | -3,3 | -1,0 | ATP-binding cassette, sub-family A (ABC1), member 11, pseudogene (ABCA11P), non-coding RNA [NR_002451] |
| LOC400604 | -4,3 | -3,3 | -1,7 | hypothetical LOC400604 (LOC400604), non-coding RNA [NR_038458] |
| RGNEF | -1,4 | -3,3 | -1,1 | 190 kDa guanine nucleotide exchange factor (RGNEF), transcript variant 1, mRNA [NM_001080479] |
| EIF3H | -2,2 | -3,3 | -4,6 | eukaryotic translation initiation factor 3, subunit H [Source:HGNC Symbol |
| NEBL | -1,8 | -3,3 | -5,3 | nebulette (NEBL), transcript variant 1, mRNA [NM_006393] |
| GRAMD4 | -1,9 | -3,3 | -1,0 | GRAM domain containing 4 (GRAMD4), mRNA [NM_015124] |
| SYTL5 | -1,5 | -3,3 | -1,6 | synaptotagmin-like 5 (SYTL5), transcript variant 2, mRNA [NM_001163335] |
| MOBKL2B | -1,7 | -3,2 | -2,2 | MOB1, Mps One Binder kinase activator-like 2B (yeast) (MOBKL2B), mRNA [NM_024761] |
| ERMAP | -2,9 | -3,2 | -1,5 | erythroblast membrane-associated protein (Scianna blood group) (ERMAP), transcript variant 1, mRNA [NM_001017922] |
| ANK3 | -1,4 | -3,2 | -2,9 | ankyrin 3, node of Ranvier (ankyrin G) (ANK3), transcript variant 1, mRNA [NM_020987] |
| SEC31B | -1,1 | -3,2 | -1,6 | SEC31 homolog B (S. cerevisiae) (SEC31B), mRNA [NM_015490] |
| CCNA1 | -2,3 | -3,2 | -1,9 | cyclin A1 (CCNA1), transcript variant 1, mRNA [NM_003914] |
| GSK3B | -1,3 | -3,2 | -1,1 | glycogen synthase kinase 3 beta [Source:HGNC Symbol |
| ELL2 | -1,6 | -3,2 | -1,9 | elongation factor, RNA polymerase II, 2 (ELL2), mRNA [NM_012081] |
| LCLAT1 | -1,5 | -3,2 | -4,9 | lysocardiolipin acyltransferase 1 [Source:HGNC Symbol |
| NAIP | -1,7 | -3,2 | -1,7 | NLR family, apoptosis inhibitory protein (NAIP), transcript variant 1, mRNA [NM_004536] |
| C1RL | -1,3 | -3,2 | -1,6 | complement component 1, r subcomponent-like (C1RL), mRNA [NM_016546] |
| PAIP2B | -2,3 | -3,2 | -4,0 | poly(A) binding protein interacting protein 2B (PAIP2B), mRNA [NM_020459] |
| TRMT1L | -1,9 | -3,1 | -1,4 | TRM1 tRNA methyltransferase 1-like (TRMT1L), transcript variant 1, mRNA [NM_030934] |
| PCSK1 | -1,9 | -3,1 | -1,9 | proprotein convertase subtilisin/kexin type 1 (PCSK1), transcript variant 1, mRNA [NM_000439] |
| LGR6 | -1,4 | -3,1 | -1,7 | leucine-rich repeat containing G protein-coupled receptor 6 (LGR6), transcript variant 1, mRNA [NM_001017403] |
| GREM2 | -1,8 | -3,1 | -2,1 | gremlin 2 (GREM2), mRNA [NM_022469] |
| ZNF160 | -1,2 | -3,1 | -1,3 | mRNA for FLJ00032 protein, partial cds. [AK024442] |
| LOC151009 | -2,7 | -3,1 | -1,5 | hypothetical LOC151009 (LOC151009), non-coding RNA [NR_027244] |
| PDE1A | -2,3 | -3,1 | -2,7 | phosphodiesterase 1A, calmodulin-dependent (PDE1A), transcript variant 2, mRNA [NM_001003683] |
| SPINK13 | 1,1 | -3,1 | -2,4 | serine peptidase inhibitor, Kazal type 13 (putative) (SPINK13), mRNA [NM_001040129] |
| ACOX3 | -1,5 | -3,1 | -1,2 | acyl-CoA oxidase 3, pristanoyl (ACOX3), transcript variant 1, mRNA [NM_003501] |
| MTHFR | -2,2 | -3,1 | -1,3 | methylenetetrahydrofolate reductase (NAD(P)H) (MTHFR), mRNA [NM_005957] |
| ZNF181 | -1,5 | -3,1 | 1,1 | zinc finger protein 181 (ZNF181), transcript variant 1, mRNA [NM_001029997] |
| SLC2A11 | -1,8 | -3,1 | -1,1 | solute carrier family 2 (facilitated glucose transporter), member 11 (SLC2A11), transcript variant 1, mRNA [NM_030807] |
| USP47 | -2,2 | -3,0 | -3,8 | ubiquitin specific peptidase 47 (USP47), mRNA [NM_017944] |
| TFDP2 | -1,5 | -3,0 | -1,5 | transcription factor Dp-2 (E2F dimerization partner 2) (TFDP2), transcript variant 1, mRNA [NM_001178138] |
| LOC100507100 | -1,4 | -3,0 | -1,5 | PREDICTED: hypothetical LOC100507100 (LOC100507100), partial miscRNA [XR_108691] |
| HCFC1 | -2,6 | -3,0 | -2,9 | host cell factor C1 (VP16-accessory protein) (HCFC1), mRNA [NM_005334] |
| PDGFRB | -2,2 | -3,0 | -1,4 | platelet-derived growth factor receptor, beta polypeptide (PDGFRB), mRNA [NM_002609] |
| KIAA1958 | 1,1 | -3,0 | -3,0 | KIAA1958 [Source:HGNC Symbol |
| SORBS2 | -2,3 | -3,0 | -1,8 | sorbin and SH3 domain containing 2 (SORBS2), transcript variant 2, mRNA [NM_021069] |
| MTR | -1,9 | -3,0 | -1,5 | 5-methyltetrahydrofolate-homocysteine methyltransferase (MTR), mRNA [NM_000254] |
| TMEM187 | -1,5 | -3,0 | -1,4 | transmembrane protein 187 (TMEM187), mRNA [NM_003492] |
| SIPA1L3 | -3,2 | -3,0 | -1,1 | signal-induced proliferation-associated 1 like 3 (SIPA1L3), mRNA [NM_015073] |
| GNAZ | -2,6 | -3,0 | 1,3 | guanine nucleotide binding protein (G protein), alpha z polypeptide (GNAZ), mRNA [NM_002073] |
| FBXO36 | -1,6 | -2,9 | -1,4 | F-box protein 36 (FBXO36), mRNA [NM_174899] |
| PSMD9 | -2,7 | -2,9 | -1,7 | proteasome (prosome, macropain) 26S subunit, non-ATPase, 9 (PSMD9), mRNA [NM_002813] |
| ELP2 | -1,6 | -2,9 | -1,8 | elongation protein 2 homolog (S. cerevisiae) [Source:HGNC Symbol |
| HAUS5 | -1,1 | -2,9 | -1,8 | HAUS augmin-like complex, subunit 5 (HAUS5), mRNA [NM_015302] |
| ZBTB1 | -1,0 | -2,9 | -1,4 | zinc finger and BTB domain containing 1 (ZBTB1), transcript variant 2, mRNA [NM_014950] |
| NEK9 | -2,5 | -2,9 | -4,0 | NIMA (never in mitosis gene a)- related kinase 9 (NEK9), mRNA [NM_033116] |
| SCAI | -1,4 | -2,9 | -1,7 | suppressor of cancer cell invasion (SCAI), transcript variant 1, mRNA [NM_173690] |
| LMLN | -1,5 | -2,9 | -1,2 | leishmanolysin-like (metallopeptidase M8 family) (LMLN), transcript variant 1, mRNA [NM_001136049] |
| ADRA2A | -4,0 | -2,9 | -2,2 | adrenergic, alpha-2A-, receptor (ADRA2A), mRNA [NM_000681] |
| PCM1 | -1,7 | -2,9 | -2,7 | pericentriolar material 1 (PCM1), mRNA [NM_006197] |
| LOC253039 | -3,0 | -2,9 | -1,3 | hypothetical LOC253039 (LOC253039), non-coding RNA [NR_024408] |
| LRRC20 | -2,4 | -2,9 | -1,0 | leucine rich repeat containing 20 (LRRC20), transcript variant 3, mRNA [NM_018205] |

**Supplementary Table 3** Pathways analysis of upregulated genes (fold change≥2, p≤0.05) with respect to control cells at A) t_0_ , B) t_3_ and C) t_6_

**Supplementary Table 4** List of transcription factors modulated in host cells by *T. cruzi* infection
